# Supplementary material for: Autoxidation of 4-Hydrazinylquinolin-2(1H)-one; Synthesis of Pyridazino[4,3-c:5,6-c′]diquinoline-6,7(5H,8H)-diones
Source: Molecules. 2022 Mar 25;27(7):2125. doi: 10.3390/molecules27072125 (PMC9000902; doi:10.3390/molecules27072125)
Supplement: Supplementary file 1 [file molecules-27-02125-s001.zip › molecules-1613746-supplementary.pdf]

Supporting Information

Sara M. Mostafa[a], Ashraf A. Aly, [a]* Alaa A. Hassan[a], Esraa M. Osman[a], Stefan Bräse,[bc]* Martin Nieger[d] M. A. A. Ibrahim,[a] and Asmaa H. Mohamed[a]

[a] *Department of Chemistry, Faculty of Science, Minia University, 61519 Minia, Egypt.*

[b] *Institute of Organic Chemistry, Karlsruhe Institute of Technology, 76131 Karlsruhe, Germany,*

[c] *Institute of Biological and Chemical Systems (IBCS-FMS), Karlsruhe Institute of Technology, Eggenstein-Leopoldshafen, Germany.*

[d] *Department of Chemistry, University of Helsinki, P.O. Box55 (A. I. Virtasenaukio I), 00014, Helsinki, Finland.*

The Table of Contents

Title, author's name, address and Table of contents	S1
Crystal structure determination of **3a**	S1
X-ray diagrams and tables of compound **3a**	S2-S13
^{1}H, ^{13}C NMR and Mass spectra of compound **3a-g**	S14-S22
Cartesian coordinates of the compound **3a** used in DFT calculation	S23-S24

Crystal Structure Determination

Single crystals were obtained by recrystallization from DMF/water. The single crystal X-ray diffraction study of **3a** was carried out on an Bruker D8 VENTURE diffractometer with PhotonII CPAD detector at 298 K using Cu $K\alpha$ radiation (λ = 1.54178 Å). Dual space methods (SHELXT) [1] were used for structure solution and refinement was carried out using SHELXL [2] (full-matrix least-squares on F^2). Hydrogen atoms were localized by difference electron density determination and refined using a riding model (H(N) free). A Semi-empirical absorption correction was applied.

3a: Orange crystals (*sb1473_hy*): $C_{22}H_{18}N_4O_2$, M_r = 370.40 g mol^{-1}, size 0.20 × 0.12 × 0.04 mm, Orthorhombic, *Pca2$_1$ (no.29)*, a = 21.1937 (4) Å, b = 9.2208 (2) Å, c = 17.9646 (3) Å, V = 3510.69 (12) Å^3, Z = 8, D_{calcd} = 1.402 Mg m^{-3}, F(000) = 1552, μ = 0.75 mm^{-1}, T = 298 K, 36156 measured reflection (2θ_{max} = 144.4^0), 6865 independent [R_{int} = 0.057], 506 parameters, 1 restraint, R_1 [for 6684 $I > 2\sigma(1)$] = 0.042, wR^2 (for all data) = 0.115, S = 1.02, largest diff. peak and hole = 0.34 eÅ^{-3}/-0.22 eÅ^{-3}.

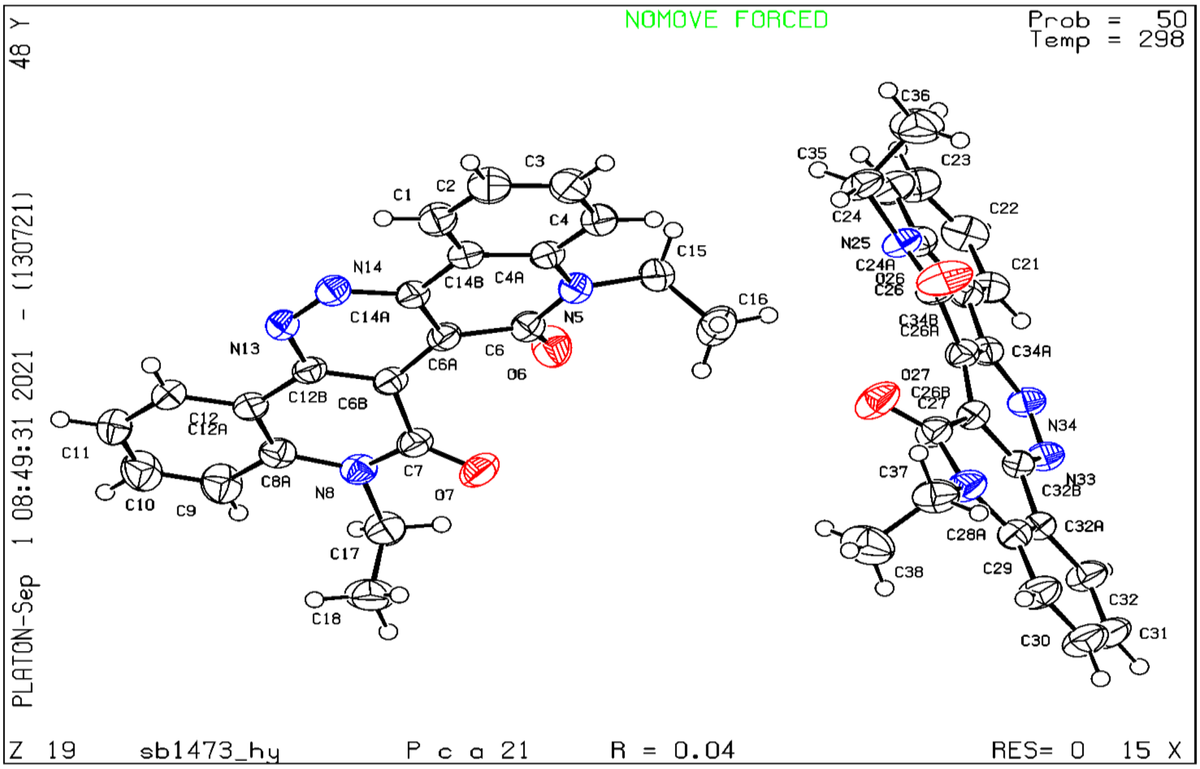

Figure S1: ORTEP diagram of compound **3a**

Supporting Information

CCDC contains the supplementary crystallographic data for this paper. These data can be obtained free of charge from The Cambridge Crystallographic Data Centre *via* www.ccdc.cam.ac.uk/data_request/cif

Table S1:Crystal data of **3a**

$C_{22}H_{18}N_4O_2$	D_x = 1.402 Mg m^{-3}
M_r = 370.40	Cu $K\alpha$ radiation, λ = 1.54178 Å
Orthorhombic, $Pca2_1$ (no.29)	Cell parameters from 9231 reflections
a = 21.1937 (4) Å	θ = 4.1–72.0°
b = 9.2208 (2) Å	μ = 0.75 mm^{-1}
c = 17.9646 (3) Å	T = 298 K
V = 3510.69 (12) Å^3	Blocks, orange
Z = 8	0.20 × 0.12 × 0.04 mm
F(000) = 1552	

Table S2: Data collection of **3a**

Bruker D8 VENTURE diffractometer with PhotonII CPAD detector	6684 reflections with $I > 2\sigma(I)$
Radiation source: INCOATEC microfocus sealed tube	R_{int} = 0.057
rotation in ϕ and ω, 1°, shutterless scans	θ_{max} = 72.2°, θ_{min} = 4.2°
Absorption correction: multi-scan *SADABS* (Sheldrick, 2014)	h = -25→26
T_{min} = 0.721, T_{max} = 0.971	k = -11→11
36156 measured reflections	l = -22→22
6865 independent reflections	

Table S3: Refinement of **3a**

Refinement on F^2	Hydrogen site location: inferred from neighbouring sites
Least-squares matrix: full	H-atom parameters constrained
$R[F^2 > 2\sigma(F^2)]$ = 0.042	$w = 1/[\sigma^2(F_o^2) + (0.0823P)^2 + 0.3357P]$ where $P = (F_o^2 + 2F_c^2)/3$
$wR(F^2)$ = 0.115	$(\Delta/\sigma)_{max}$ < 0.001
S = 1.02	$\Delta\rangle_{max}$ = 0.34 e Å^{-3}
6865 reflections	$\Delta\rangle_{min}$ = -0.22 e Å^{-3}
506 parameters	Extinction correction: *SHELXL2014*/7 (Sheldrick 2014), $Fc^*=kFc[1+0.001xFc^2\lambda^3/\sin(2\theta)]^{-1/4}$
1 restraint	Extinction coefficient: 0.0033 (5)
Primary atom site location: dual	Absolute structure: Flack x determined using 3109 quotients [(I+)-(I-)]/[(I+)+(I-)] (Parsons, Flack and Wagner, Acta Cryst. B69 (2013) 249-259).
Secondary atom site location: difference Fourier map	Absolute structure parameter: -0.05 (11)

Computing details

Data collection: *APEX3* (Bruker AXS Inc., 2019); cell refinement: *APEX3* (Bruker AXS Inc., 2019); data reduction: *SAINT* (Bruker AXS Inc., 2019); program(s) used to solve structure: SHELXT (Sheldrick, 2015); program(s) used to refine structure: *SHELXL2014*/7 (Sheldrick, 2014); software used to prepare material for publication: *publCIF* (Westrip, 2010).

Special details

Experimental. dx = 40 mm, 1 deg., 7+1 runs, 1623 frames, 20/60 sec./frame
Geometry. All esds (except the esd in the dihedral angle between two l.s. planes) are estimated using the full covariance matrix. The cell esds are taken into account individually in the estimation of

esds in distances, angles and torsion angles; correlations between esds in cell parameters are only used when they are defined by crystal symmetry. An approximate (isotropic) treatment of cell esds is used for estimating esds involving l.s. planes.

Table S4: Fractional atomic coordinates and isotropic or equivalent isotropic displacement parameters (Å^2) for 3a

	X	*y*	*z*	U_{iso}*/U_{eq}
C1	0.23494 (14)	0.5929 (3)	0.84849 (14)	0.0474 (6)
H1	0.2138	0.5412	0.8853	0.057*
C2	0.28285 (15)	0.6853 (3)	0.86815 (15)	0.0543 (7)
H2	0.2947	0.6950	0.9178	0.065*
C3	0.31348 (14)	0.7639 (3)	0.81319 (17)	0.0520 (7)
H3	0.3459	0.8268	0.8264	0.062*
C4	0.29664 (13)	0.7505 (3)	0.73918 (16)	0.0443 (5)
H4	0.3175	0.8047	0.7031	0.053*
C4A	0.24837 (11)	0.6558 (2)	0.71859 (13)	0.0359 (5)
N5	0.22907 (9)	0.6439 (2)	0.64395 (11)	0.0387 (4)
C6	0.18389 (12)	0.5467 (3)	0.61944 (13)	0.0389 (5)
O6	0.16223 (10)	0.5532 (2)	0.55655 (10)	0.0561 (5)
C6A	0.15961 (11)	0.4431 (2)	0.67592 (12)	0.0344 (5)
C6B	0.12010 (10)	0.3258 (2)	0.65930 (12)	0.0337 (5)
C7	0.11905 (11)	0.2506 (3)	0.58608 (13)	0.0374 (5)
O7	0.16339 (9)	0.2561 (2)	0.54270 (11)	0.0527 (5)
N8	0.06651 (9)	0.1674 (2)	0.57177 (11)	0.0375 (4)
C8A	0.02501 (11)	0.1215 (3)	0.62793 (14)	0.0376 (5)
C9	-0.02635 (13)	0.0307 (3)	0.61168 (17)	0.0525 (7)
H9	-0.0353	0.0064	0.5626	0.063*
C10	-0.06345 (13)	-0.0223 (4)	0.66819 (19)	0.0571 (7)
H10	-0.0969	-0.0835	0.6567	0.068*
C11	-0.05206 (13)	0.0134 (3)	0.74172 (18)	0.0507 (6)
H11	-0.0774	-0.0243	0.7793	0.061*
C12	-0.00255 (12)	0.1058 (3)	0.75882 (14)	0.0420 (5)
H12	0.0053	0.1304	0.8082	0.050*
C12A	0.03573 (11)	0.1624 (2)	0.70232 (13)	0.0357 (5)
C12B	0.08528 (10)	0.2666 (2)	0.71784 (13)	0.0340 (5)
N13	0.09417 (10)	0.3063 (2)	0.78974 (12)	0.0404 (4)
N14	0.13790 (11)	0.4009 (2)	0.80667 (11)	0.0407 (5)
C14A	0.16972 (11)	0.4714 (2)	0.75153 (13)	0.0350 (5)
C14B	0.21738 (11)	0.5755 (2)	0.77385 (13)	0.0363 (5)

C15	0.24985 (13)	0.7516 (3)	0.58839 (15)	0.0441 (5)
H15A	0.2586	0.8426	0.6134	0.053*
H15B	0.2159	0.7684	0.5533	0.053*
C16	0.30796 (16)	0.7043 (4)	0.54628 (18)	0.0595 (7)
H16A	0.3194	0.7779	0.5110	0.089*
H16B	0.3421	0.6895	0.5806	0.089*
H16C	0.2993	0.6153	0.5204	0.089*
C17	0.06431 (13)	0.0990 (3)	0.49717 (13)	0.0454 (6)
H17A	0.0851	0.1620	0.4616	0.054*
H17B	0.0206	0.0886	0.4819	0.054*
C18	0.0958 (2)	-0.0484 (3)	0.4959 (2)	0.0683 (9)
H18A	0.0933	-0.0884	0.4467	0.102*
H18B	0.1393	-0.0384	0.5100	0.102*
H18C	0.0749	-0.1119	0.5303	0.102*
C21	0.25246 (12)	1.1259 (3)	0.22702 (14)	0.0407 (5)
H21	0.2622	1.1020	0.1781	0.049*
C22	0.20234 (13)	1.2168 (3)	0.24152 (18)	0.0504 (6)
H22	0.1790	1.2561	0.2026	0.060*
C23	0.18707 (14)	1.2492 (3)	0.31447 (19)	0.0555 (7)
H23	0.1522	1.3076	0.3242	0.067*
C24	0.22214 (13)	1.1972 (3)	0.37303 (16)	0.0499 (6)
H24	0.2112	1.2209	0.4217	0.060*
C24A	0.27449 (11)	1.1082 (3)	0.35919 (13)	0.0373 (5)
N25	0.31436 (10)	1.0631 (2)	0.41753 (11)	0.0388 (4)
C26	0.37076 (11)	0.9921 (3)	0.40456 (12)	0.0382 (5)
O26	0.41412 (9)	0.9983 (3)	0.44840 (11)	0.0573 (5)
C26A	0.37492 (11)	0.9135 (2)	0.33269 (12)	0.0339 (5)
C26B	0.41652 (10)	0.7986 (2)	0.31820 (12)	0.0337 (4)
C27	0.44531 (12)	0.7072 (3)	0.37716 (13)	0.0395 (5)
O27	0.42576 (10)	0.7038 (3)	0.44070 (11)	0.0574 (5)
N28	0.49696 (11)	0.6249 (2)	0.35563 (12)	0.0448 (5)
C28A	0.50990 (11)	0.5933 (2)	0.28086 (14)	0.0386 (5)
C29	0.55826 (14)	0.4966 (3)	0.26122 (16)	0.0508 (6)
H29	0.5830	0.4545	0.2981	0.061*
C30	0.56913 (15)	0.4641 (4)	0.18774 (18)	0.0567 (7)
H30	0.6013	0.3996	0.1758	0.068*
C31	0.53376 (15)	0.5242 (3)	0.13121 (16)	0.0541 (7)
H31	0.5416	0.4996	0.0819	0.065*
C32	0.48653 (13)	0.6215 (3)	0.14899 (14)	0.0453 (6)
H32	0.4628	0.6638	0.1112	0.054*
C32A	0.47388 (11)	0.6573 (2)	0.22359 (13)	0.0349 (5)

C32B	0.42625 (10)	0.7637 (2)	0.24345 (12)	0.0329 (4)
N33	0.39397 (9)	0.8266 (2)	0.18726 (11)	0.0376 (4)
N34	0.34959 (9)	0.9216 (2)	0.20167 (11)	0.0379 (4)
C34A	0.33965 (10)	0.9657 (2)	0.27273 (12)	0.0330 (4)
C34B	0.28880 (10)	1.0690 (2)	0.28525 (13)	0.0343 (5)
C35	0.30535 (14)	1.1196 (3)	0.49395 (14)	0.0483 (6)
H35A	0.3269	1.0561	0.5287	0.058*
H35B	0.2607	1.1175	0.5059	0.058*
C36	0.3295 (2)	1.2711 (4)	0.5041 (2)	0.0734 (10)
H36A	0.3223	1.3015	0.5545	0.110*
H36B	0.3739	1.2737	0.4935	0.110*
H36C	0.3077	1.3351	0.4707	0.110*
C37	0.53214 (15)	0.5515 (3)	0.41664 (16)	0.0530 (7)
H37A	0.5273	0.6069	0.4622	0.064*
H37B	0.5767	0.5493	0.4043	0.064*
C38	0.5093 (2)	0.3994 (4)	0.4294 (2)	0.0725 (10)
H38A	0.5332	0.3561	0.4691	0.109*
H38B	0.4655	0.4012	0.4426	0.109*
H38C	0.5148	0.3437	0.3848	0.109*

Table S5: Atomic displacement parameters (Å^2) for 3a

	U^{11}	U^{22}	U^{33}	U^{12}	U^{13}	U^{23}
C1	0.0578 (15)	0.0511 (14)	0.0333 (12)	-0.0088 (11)	0.0014 (11)	-0.0049 (11)
C2	0.0619 (16)	0.0613 (16)	0.0399 (14)	-0.0136 (13)	-0.0044 (12)	-0.0106 (12)
C3	0.0487 (14)	0.0550 (15)	0.0524 (16)	-0.0134 (11)	0.0017 (12)	-0.0127 (13)
C4	0.0432 (12)	0.0432 (12)	0.0466 (13)	-0.0051 (10)	0.0057 (11)	-0.0041 (11)
C4A	0.0391 (10)	0.0340 (10)	0.0344 (11)	0.0029 (9)	0.0044 (9)	-0.0024 (9)
N5	0.0442 (10)	0.0393 (10)	0.0326 (9)	-0.0020 (8)	0.0040 (8)	0.0007 (8)
C6	0.0444 (12)	0.0411 (12)	0.0310 (11)	-0.0025 (9)	0.0044 (9)	-0.0009 (9)
O6	0.0713 (13)	0.0645 (12)	0.0324 (9)	-0.0169 (10)	-0.0057 (9)	0.0064 (8)
C6A	0.0365 (10)	0.0369 (11)	0.0297 (11)	0.0018 (9)	0.0037 (8)	-0.0018 (9)
C6B	0.0318 (10)	0.0366 (11)	0.0326 (11)	0.0018 (8)	0.0028 (8)	-0.0042 (9)
C7	0.0382 (11)	0.0409 (11)	0.0330 (11)	0.0007 (9)	0.0025 (9)	-0.0046 (9)
O7	0.0477 (10)	0.0674 (12)	0.0430 (10)	-0.0103 (8)	0.0135 (8)	-0.0181 (9)
N8	0.0388 (9)	0.0409 (10)	0.0327 (9)	-0.0014 (7)	-0.0023 (8)	-0.0040 (8)
C8A	0.0344 (11)	0.0372 (11)	0.0411 (12)	0.0028 (9)	-0.0017 (9)	-0.0006 (9)
C9	0.0424 (13)	0.0634 (17)	0.0518 (15)	-0.0129 (12)	-0.0076 (11)	-0.0021 (13)
C10	0.0426 (13)	0.0616 (17)	0.0669 (19)	-0.0146 (12)	-0.0034 (13)	-0.0019 (15)
C11	0.0427 (12)	0.0507 (14)	0.0586 (16)	-0.0056 (11)	0.0134 (12)	0.0010 (12)
C12	0.0426 (12)	0.0398 (12)	0.0434 (13)	0.0011 (9)	0.0115 (10)	-0.0024 (10)

C12A	0.0330 (10)	0.0339 (10)	0.0402 (12)	0.0046 (8)	0.0038 (9)	-0.0015 (9)
C12B	0.0347 (10)	0.0332 (10)	0.0341 (11)	0.0044 (8)	0.0036 (9)	-0.0028 (9)
N13	0.0467 (11)	0.0399 (10)	0.0346 (10)	-0.0065 (8)	0.0082 (8)	-0.0033 (8)
N14	0.0501 (11)	0.0416 (10)	0.0304 (9)	-0.0054 (8)	0.0040 (8)	-0.0030 (8)
C14A	0.0401 (11)	0.0333 (10)	0.0317 (10)	0.0006 (8)	0.0034 (9)	-0.0019 (9)
C14B	0.0396 (11)	0.0347 (10)	0.0345 (11)	0.0004 (9)	0.0030 (9)	-0.0044 (9)
C15	0.0527 (13)	0.0394 (12)	0.0403 (12)	-0.0017 (10)	0.0051 (11)	0.0045 (10)
C16	0.0615 (17)	0.0703 (18)	0.0466 (15)	0.0047 (14)	0.0157 (13)	0.0070 (14)
C17	0.0561 (14)	0.0500 (14)	0.0301 (11)	-0.0063 (11)	-0.0076 (10)	-0.0033 (10)
C18	0.106 (3)	0.0475 (15)	0.0514 (16)	0.0033 (16)	0.0055 (18)	-0.0120 (13)
C21	0.0410 (11)	0.0412 (12)	0.0397 (12)	0.0004 (10)	-0.0070 (10)	-0.0040 (10)
C22	0.0430 (13)	0.0502 (14)	0.0580 (16)	0.0054 (11)	-0.0158 (12)	-0.0016 (13)
C23	0.0428 (14)	0.0599 (16)	0.0639 (18)	0.0157 (11)	-0.0043 (13)	-0.0077 (14)
C24	0.0414 (13)	0.0590 (16)	0.0492 (15)	0.0098 (11)	0.0036 (11)	-0.0065 (12)
C24A	0.0339 (11)	0.0404 (12)	0.0377 (12)	0.0006 (8)	0.0000 (9)	-0.0031 (9)
N25	0.0418 (10)	0.0452 (10)	0.0294 (10)	0.0063 (8)	0.0025 (7)	-0.0059 (8)
C26	0.0422 (12)	0.0429 (11)	0.0295 (11)	0.0063 (9)	-0.0031 (9)	-0.0033 (9)
O26	0.0548 (10)	0.0761 (13)	0.0412 (9)	0.0216 (10)	-0.0171 (9)	-0.0172 (9)
C26A	0.0345 (10)	0.0369 (11)	0.0303 (11)	-0.0001 (8)	0.0004 (8)	-0.0024 (9)
C26B	0.0337 (10)	0.0368 (11)	0.0305 (11)	0.0006 (9)	0.0023 (8)	-0.0007 (9)
C27	0.0434 (12)	0.0417 (12)	0.0332 (11)	0.0072 (9)	0.0035 (10)	0.0018 (9)
O27	0.0678 (12)	0.0703 (13)	0.0341 (9)	0.0237 (10)	0.0125 (9)	0.0081 (9)
N28	0.0522 (12)	0.0470 (11)	0.0351 (10)	0.0126 (9)	0.0034 (9)	0.0020 (9)
C28A	0.0407 (12)	0.0366 (11)	0.0385 (12)	0.0023 (9)	0.0065 (10)	0.0006 (10)
C29	0.0510 (14)	0.0541 (15)	0.0474 (15)	0.0180 (12)	0.0047 (12)	-0.0006 (12)
C30	0.0557 (16)	0.0598 (16)	0.0545 (16)	0.0205 (13)	0.0135 (13)	-0.0050 (13)
C31	0.0610 (16)	0.0582 (16)	0.0430 (14)	0.0130 (13)	0.0139 (12)	-0.0087 (13)
C32	0.0515 (14)	0.0493 (14)	0.0350 (12)	0.0075 (11)	0.0046 (10)	-0.0055 (10)
C32A	0.0374 (10)	0.0336 (10)	0.0338 (11)	-0.0023 (8)	0.0054 (9)	-0.0027 (9)
C32B	0.0349 (10)	0.0320 (10)	0.0317 (10)	-0.0037 (8)	0.0029 (9)	-0.0026 (8)
N33	0.0404 (10)	0.0404 (10)	0.0319 (9)	0.0024 (8)	-0.0015 (8)	-0.0054 (8)
N34	0.0413 (10)	0.0400 (10)	0.0325 (10)	0.0018 (8)	-0.0019 (8)	-0.0044 (8)
C34A	0.0337 (10)	0.0339 (10)	0.0313 (10)	-0.0024 (8)	-0.0028 (8)	-0.0018 (8)
C34B	0.0320 (10)	0.0349 (10)	0.0360 (11)	-0.0023 (8)	-0.0028 (9)	-0.0014 (9)
C35	0.0600 (15)	0.0544 (14)	0.0304 (11)	0.0129 (12)	0.0074 (11)	-0.0042 (10)
C36	0.109 (3)	0.0595 (18)	0.0522 (17)	-0.0013 (18)	-0.0027 (18)	-0.0151 (15)
C37	0.0593 (15)	0.0532 (15)	0.0467 (14)	0.0066 (13)	-0.0046 (12)	-0.0063 (12)
C38	0.106 (3)	0.0536 (17)	0.0577 (18)	0.0020 (18)	-0.0133 (18)	0.0034 (15)

Table S6: Geometric parameters (Å, º) for 3a

C1—C2	1.371 (4)	C21—C22	1.378 (4)
C1—C14B	1.401 (3)	C21—C34B	1.401 (3)
C1—H1	0.9300	C21—H21	0.9300
C2—C3	1.386 (4)	C22—C23	1.383 (4)
C2—H2	0.9300	C22—H22	0.9300
C3—C4	1.382 (4)	C23—C24	1.374 (4)
C3—H3	0.9300	C23—H23	0.9300
C4—C4A	1.395 (4)	C24—C24A	1.402 (3)
C4—H4	0.9300	C24—H24	0.9300
C4A—C14B	1.402 (3)	C24A—N25	1.409 (3)
C4A—N5	1.406 (3)	C24A—C34B	1.410 (3)
N5—C6	1.383 (3)	N25—C26	1.383 (3)
N5—C15	1.475 (3)	N25—C35	1.480 (3)
C6—O6	1.221 (3)	C26—O26	1.212 (3)
C6—C6A	1.486 (3)	C26—C26A	1.483 (3)
C6A—C14A	1.400 (3)	C26A—C34A	1.397 (3)
C6A—C6B	1.400 (3)	C26A—C26B	1.402 (3)
C6B—C12B	1.396 (3)	C26B—C32B	1.396 (3)
C6B—C7	1.487 (3)	C26B—C27	1.485 (3)
C7—O7	1.222 (3)	C27—O27	1.215 (3)
C7—N8	1.377 (3)	C27—N28	1.387 (3)
N8—C8A	1.404 (3)	N28—C28A	1.402 (3)
N8—C17	1.482 (3)	N28—C37	1.488 (4)
C8A—C9	1.404 (3)	C28A—C29	1.403 (3)
C8A—C12A	1.407 (3)	C28A—C32A	1.411 (3)
C9—C10	1.374 (4)	C29—C30	1.373 (4)
C9—H9	0.9300	C29—H29	0.9300
C10—C11	1.382 (5)	C30—C31	1.378 (5)
C10—H10	0.9300	C30—H30	0.9300
C11—C12	1.386 (4)	C31—C32	1.382 (4)
C11—H11	0.9300	C31—H31	0.9300
C12—C12A	1.400 (3)	C32—C32A	1.406 (3)
C12—H12	0.9300	C32—H32	0.9300
C12A—C12B	1.451 (3)	C32A—C32B	1.452 (3)
C12B—N13	1.356 (3)	C32B—N33	1.350 (3)
N13—N14	1.308 (3)	N33—N34	1.311 (3)
N14—C14A	1.363 (3)	N34—C34A	1.356 (3)
C14A—C14B	1.450 (3)	C34A—C34B	1.455 (3)
C15—C16	1.510 (4)	C35—C36	1.499 (5)
C15—H15A	0.9700	C35—H35A	0.9700

C15—H15B	0.9700	C35—H35B	0.9700
C16—H16A	0.9600	C36—H36A	0.9600
C16—H16B	0.9600	C36—H36B	0.9600
C16—H16C	0.9600	C36—H36C	0.9600
C17—C18	1.515 (4)	C37—C38	1.501 (5)
C17—H17A	0.9700	C37—H37A	0.9700
C17—H17B	0.9700	C37—H37B	0.9700
C18—H18A	0.9600	C38—H38A	0.9600
C18—H18B	0.9600	C38—H38B	0.9600
C18—H18C	0.9600	C38—H38C	0.9600
C2—C1—C14B	121.0 (3)	C22—C21—C34B	120.7 (2)
C2—C1—H1	119.5	C22—C21—H21	119.7
C14B—C1—H1	119.5	C34B—C21—H21	119.7
C1—C2—C3	119.2 (3)	C21—C22—C23	119.4 (3)
C1—C2—H2	120.4	C21—C22—H22	120.3
C3—C2—H2	120.4	C23—C22—H22	120.3
C4—C3—C2	121.2 (3)	C24—C23—C22	121.6 (2)
C4—C3—H3	119.4	C24—C23—H23	119.2
C2—C3—H3	119.4	C22—C23—H23	119.2
C3—C4—C4A	120.0 (3)	C23—C24—C24A	119.8 (3)
C3—C4—H4	120.0	C23—C24—H24	120.1
C4A—C4—H4	120.0	C24A—C24—H24	120.1
C4—C4A—C14B	119.1 (2)	C24—C24A—N25	121.0 (2)
C4—C4A—N5	121.0 (2)	C24—C24A—C34B	119.2 (2)
C14B—C4A—N5	119.8 (2)	N25—C24A—C34B	119.8 (2)
C6—N5—C4A	123.8 (2)	C26—N25—C24A	122.19 (19)
C6—N5—C15	115.3 (2)	C26—N25—C35	115.7 (2)
C4A—N5—C15	120.4 (2)	C24A—N25—C35	120.6 (2)
O6—C6—N5	121.6 (2)	O26—C26—N25	121.6 (2)
O6—C6—C6A	122.2 (2)	O26—C26—C26A	122.9 (2)
N5—C6—C6A	116.0 (2)	N25—C26—C26A	115.4 (2)
C14A—C6A—C6B	116.3 (2)	C34A—C26A—C26B	117.0 (2)
C14A—C6A—C6	119.3 (2)	C34A—C26A—C26	118.11 (19)
C6B—C6A—C6	124.0 (2)	C26B—C26A—C26	124.6 (2)
C12B—C6B—C6A	117.2 (2)	C32B—C26B—C26A	116.5 (2)
C12B—C6B—C7	118.4 (2)	C32B—C26B—C27	119.6 (2)
C6A—C6B—C7	123.9 (2)	C26A—C26B—C27	123.7 (2)
O7—C7—N8	121.8 (2)	O27—C27—N28	121.1 (2)
O7—C7—C6B	122.2 (2)	O27—C27—C26B	123.0 (2)
N8—C7—C6B	115.9 (2)	N28—C27—C26B	115.9 (2)

C7—N8—C8A	122.72 (19)	C27—N28—C28A	122.4 (2)
C7—N8—C17	115.6 (2)	C27—N28—C37	116.0 (2)
C8A—N8—C17	120.16 (19)	C28A—N28—C37	120.9 (2)
N8—C8A—C9	121.1 (2)	N28—C28A—C29	121.1 (2)
N8—C8A—C12A	120.1 (2)	N28—C28A—C32A	120.4 (2)
C9—C8A—C12A	118.8 (2)	C29—C28A—C32A	118.5 (2)
C10—C9—C8A	120.1 (3)	C30—C29—C28A	120.2 (3)
C10—C9—H9	119.9	C30—C29—H29	119.9
C8A—C9—H9	119.9	C28A—C29—H29	119.9
C9—C10—C11	121.4 (3)	C29—C30—C31	121.9 (2)
C9—C10—H10	119.3	C29—C30—H30	119.0
C11—C10—H10	119.3	C31—C30—H30	119.0
C10—C11—C12	119.3 (3)	C30—C31—C32	119.0 (3)
C10—C11—H11	120.3	C30—C31—H31	120.5
C12—C11—H11	120.3	C32—C31—H31	120.5
C11—C12—C12A	120.5 (3)	C31—C32—C32A	120.7 (3)
C11—C12—H12	119.8	C31—C32—H32	119.6
C12A—C12—H12	119.8	C32A—C32—H32	119.6
C12—C12A—C8A	119.7 (2)	C32—C32A—C28A	119.6 (2)
C12—C12A—C12B	121.8 (2)	C32—C32A—C32B	121.7 (2)
C8A—C12A—C12B	118.5 (2)	C28A—C32A—C32B	118.7 (2)
N13—C12B—C6B	122.6 (2)	N33—C32B—C26B	123.0 (2)
N13—C12B—C12A	117.6 (2)	N33—C32B—C32A	117.3 (2)
C6B—C12B—C12A	119.8 (2)	C26B—C32B—C32A	119.7 (2)
N14—N13—C12B	119.99 (19)	N34—N33—C32B	120.18 (19)
N13—N14—C14A	120.0 (2)	N33—N34—C34A	119.85 (19)
N14—C14A—C6A	122.7 (2)	N34—C34A—C26A	122.6 (2)
N14—C14A—C14B	117.3 (2)	N34—C34A—C34B	117.2 (2)
C6A—C14A—C14B	119.9 (2)	C26A—C34A—C34B	120.2 (2)
C1—C14B—C4A	119.5 (2)	C21—C34B—C24A	119.3 (2)
C1—C14B—C14A	121.7 (2)	C21—C34B—C34A	122.5 (2)
C4A—C14B—C14A	118.7 (2)	C24A—C34B—C34A	118.2 (2)
N5—C15—C16	112.8 (2)	N25—C35—C36	113.4 (2)
N5—C15—H15A	109.0	N25—C35—H35A	108.9
C16—C15—H15A	109.0	C36—C35—H35A	108.9
N5—C15—H15B	109.0	N25—C35—H35B	108.9
C16—C15—H15B	109.0	C36—C35—H35B	108.9
H15A—C15—H15B	107.8	H35A—C35—H35B	107.7
C15—C16—H16A	109.5	C35—C36—H36A	109.5
C15—C16—H16B	109.5	C35—C36—H36B	109.5
H16A—C16—H16B	109.5	H36A—C36—H36B	109.5

C15—C16—H16C	109.5	C35—C36—H36C	109.5
H16A—C16—H16C	109.5	H36A—C36—H36C	109.5
H16B—C16—H16C	109.5	H36B—C36—H36C	109.5
N8—C17—C18	112.4 (2)	N28—C37—C38	112.1 (3)
N8—C17—H17A	109.1	N28—C37—H37A	109.2
C18—C17—H17A	109.1	C38—C37—H37A	109.2
N8—C17—H17B	109.1	N28—C37—H37B	109.2
C18—C17—H17B	109.1	C38—C37—H37B	109.2
H17A—C17—H17B	107.9	H37A—C37—H37B	107.9
C17—C18—H18A	109.5	C37—C38—H38A	109.5
C17—C18—H18B	109.5	C37—C38—H38B	109.5
H18A—C18—H18B	109.5	H38A—C38—H38B	109.5
C17—C18—H18C	109.5	C37—C38—H38C	109.5
H18A—C18—H18C	109.5	H38A—C38—H38C	109.5
H18B—C18—H18C	109.5	H38B—C38—H38C	109.5
C14B—C1—C2—C3	1.1 (5)	C34B—C21—C22—C23	-1.5 (4)
C1—C2—C3—C4	-0.1 (5)	C21—C22—C23—C24	2.5 (5)
C2—C3—C4—C4A	-0.5 (5)	C22—C23—C24—C24A	-0.5 (5)
C3—C4—C4A—C14B	0.2 (4)	C23—C24—C24A—N25	174.7 (3)
C3—C4—C4A—N5	178.0 (2)	C23—C24—C24A—C34B	-2.4 (4)
C4—C4A—N5—C6	176.5 (2)	C24—C24A—N25—C26	-171.1 (3)
C14B—C4A—N5—C6	-5.8 (3)	C34B—C24A—N25—C26	6.0 (3)
C4—C4A—N5—C15	-12.7 (3)	C24—C24A—N25—C35	-5.6 (4)
C14B—C4A—N5—C15	165.0 (2)	C34B—C24A—N25—C35	171.5 (2)
C4A—N5—C6—O6	169.7 (2)	C24A—N25—C26—O26	153.5 (3)
C15—N5—C6—O6	-1.5 (3)	C35—N25—C26—O26	-12.6 (4)
C4A—N5—C6—C6A	-5.3 (3)	C24A—N25—C26—C26A	-24.3 (3)
C15—N5—C6—C6A	-176.5 (2)	C35—N25—C26—C26A	169.6 (2)
O6—C6—C6A—C14A	-158.3 (2)	O26—C26—C26A—C34A	-149.0 (3)
N5—C6—C6A—C14A	16.6 (3)	N25—C26—C26A—C34A	28.8 (3)
O6—C6—C6A—C6B	13.6 (4)	O26—C26—C26A—C26B	24.4 (4)
N5—C6—C6A—C6B	-171.5 (2)	N25—C26—C26A—C26B	-157.8 (2)
C14A—C6A—C6B—C12B	12.0 (3)	C34A—C26A—C26B—C32B	10.2 (3)
C6—C6A—C6B—C12B	-160.1 (2)	C26—C26A—C26B—C32B	-163.3 (2)
C14A—C6A—C6B—C7	-160.3 (2)	C34A—C26A—C26B—C27	-164.4 (2)
C6—C6A—C6B—C7	27.6 (3)	C26—C26A—C26B—C27	22.2 (4)
C12B—C6B—C7—O7	-150.8 (2)	C32B—C26B—C27—O27	-158.6 (3)
C6A—C6B—C7—O7	21.4 (4)	C26A—C26B—C27—O27	15.8 (4)
C12B—C6B—C7—N8	25.6 (3)	C32B—C26B—C27—N28	21.7 (3)
C6A—C6B—C7—N8	-162.1 (2)	C26A—C26B—C27—N28	-163.9 (2)

O7—C7—N8—C8A	159.1 (2)	O27—C27—N28—C28A	161.4 (3)
C6B—C7—N8—C8A	-17.3 (3)	C26B—C27—N28—C28A	-19.0 (4)
O7—C7—N8—C17	-6.6 (3)	O27—C27—N28—C37	-8.9 (4)
C6B—C7—N8—C17	176.9 (2)	C26B—C27—N28—C37	170.7 (2)
C7—N8—C8A—C9	-176.8 (2)	C27—N28—C28A—C29	-172.8 (3)
C17—N8—C8A—C9	-11.7 (3)	C37—N28—C28A—C29	-2.9 (4)
C7—N8—C8A—C12A	1.0 (3)	C27—N28—C28A—C32A	6.5 (4)
C17—N8—C8A—C12A	166.1 (2)	C37—N28—C28A—C32A	176.4 (2)
N8—C8A—C9—C10	174.8 (3)	N28—C28A—C29—C30	178.3 (3)
C12A—C8A—C9—C10	-3.0 (4)	C32A—C28A—C29—C30	-1.1 (4)
C8A—C9—C10—C11	0.9 (5)	C28A—C29—C30—C31	0.2 (5)
C9—C10—C11—C12	0.7 (5)	C29—C30—C31—C32	0.9 (5)
C10—C11—C12—C12A	-0.1 (4)	C30—C31—C32—C32A	-1.0 (5)
C11—C12—C12A—C8A	-2.0 (3)	C31—C32—C32A—C28A	0.1 (4)
C11—C12—C12A—C12B	175.9 (2)	C31—C32—C32A—C32B	177.4 (2)
N8—C8A—C12A—C12	-174.3 (2)	N28—C28A—C32A—C32	-178.4 (2)
C9—C8A—C12A—C12	3.5 (3)	C29—C28A—C32A—C32	0.9 (4)
N8—C8A—C12A—C12B	7.7 (3)	N28—C28A—C32A—C32B	4.2 (3)
C9—C8A—C12A—C12B	-174.5 (2)	C29—C28A—C32A—C32B	-176.5 (2)
C6A—C6B—C12B—N13	-8.2 (3)	C26A—C26B—C32B—N33	-5.6 (3)
C7—C6B—C12B—N13	164.6 (2)	C27—C26B—C32B—N33	169.2 (2)
C6A—C6B—C12B—C12A	169.4 (2)	C26A—C26B—C32B—C32A	173.11 (19)
C7—C6B—C12B—C12A	-17.8 (3)	C27—C26B—C32B—C32A	-12.1 (3)
C12—C12A—C12B—N13	1.0 (3)	C32—C32A—C32B—N33	0.5 (3)
C8A—C12A—C12B—N13	179.0 (2)	C28A—C32A—C32B—N33	177.86 (19)
C12—C12A—C12B—C6B	-176.7 (2)	C32—C32A—C32B—C26B	-178.3 (2)
C8A—C12A—C12B—C6B	1.2 (3)	C28A—C32A—C32B—C26B	-0.9 (3)
C6B—C12B—N13—N14	-1.8 (3)	C26B—C32B—N33—N34	-2.5 (3)
C12A—C12B—N13—N14	-179.4 (2)	C32A—C32B—N33—N34	178.8 (2)
C12B—N13—N14—C14A	7.3 (3)	C32B—N33—N34—C34A	5.5 (3)
N13—N14—C14A—C6A	-2.7 (3)	N33—N34—C34A—C26A	-0.3 (3)
N13—N14—C14A—C14B	179.7 (2)	N33—N34—C34A—C34B	-178.7 (2)
C6B—C6A—C14A—N14	-7.2 (3)	C26B—C26A—C34A—N34	-7.8 (3)
C6—C6A—C14A—N14	165.3 (2)	C26—C26A—C34A—N34	166.1 (2)
C6B—C6A—C14A—C14B	170.4 (2)	C26B—C26A—C34A—C34B	170.6 (2)
C6—C6A—C14A—C14B	-17.1 (3)	C26—C26A—C34A—C34B	-15.5 (3)
C2—C1—C14B—C4A	-1.3 (4)	C22—C21—C34B—C24A	-1.4 (3)
C2—C1—C14B—C14A	175.9 (3)	C22—C21—C34B—C34A	176.2 (2)
C4—C4A—C14B—C1	0.7 (3)	C24—C24A—C34B—C21	3.3 (3)
N5—C4A—C14B—C1	-177.1 (2)	N25—C24A—C34B—C21	-173.8 (2)
C4—C4A—C14B—C14A	-176.6 (2)	C24—C24A—C34B—C34A	-174.3 (2)

N5—C4A—C14B—C14A	5.6 (3)	N25—C24A—C34B—C34A	8.5 (3)
N14—C14A—C14B—C1	6.4 (3)	N34—C34A—C34B—C21	-2.2 (3)
C6A—C14A—C14B—C1	-171.3 (2)	C26A—C34A—C34B—C21	179.3 (2)
N14—C14A—C14B—C4A	-176.4 (2)	N34—C34A—C34B—C24A	175.3 (2)
C6A—C14A—C14B—C4A	5.9 (3)	C26A—C34A—C34B—C24A	-3.1 (3)
C6—N5—C15—C16	-93.6 (3)	C26—N25—C35—C36	89.7 (3)
C4A—N5—C15—C16	94.8 (3)	C24A—N25—C35—C36	-76.7 (3)
C7—N8—C17—C18	88.8 (3)	C27—N28—C37—C38	93.4 (3)
C8A—N8—C17—C18	-77.4 (3)	C28A—N28—C37—C38	-77.1 (4)

Table S7: Hydrogen-bond geometry (Å, º) for 3a

D—H···*A*	*D*—H	H···*A*	*D*···*A*	*D*—H···*A*
C9—H9···O26^{i}	0.93	2.31	3.204 (4)	160
C17—H17*B*···O26^{i}	0.97	2.47	3.421 (3)	167
C24—H24···O7^{ii}	0.93	2.42	3.337 (3)	168
C35—H35*B*···O7^{ii}	0.97	2.52	3.377 (3)	148

Symmetry codes: (i) *x*-1/2, -*y*+1, *z*; (ii) *x*, *y*+1, *z*.

Document origin: *publCIF* [Westrip, S. P. (2010). *J. Apply. Cryst.*, **43**, 920-925].

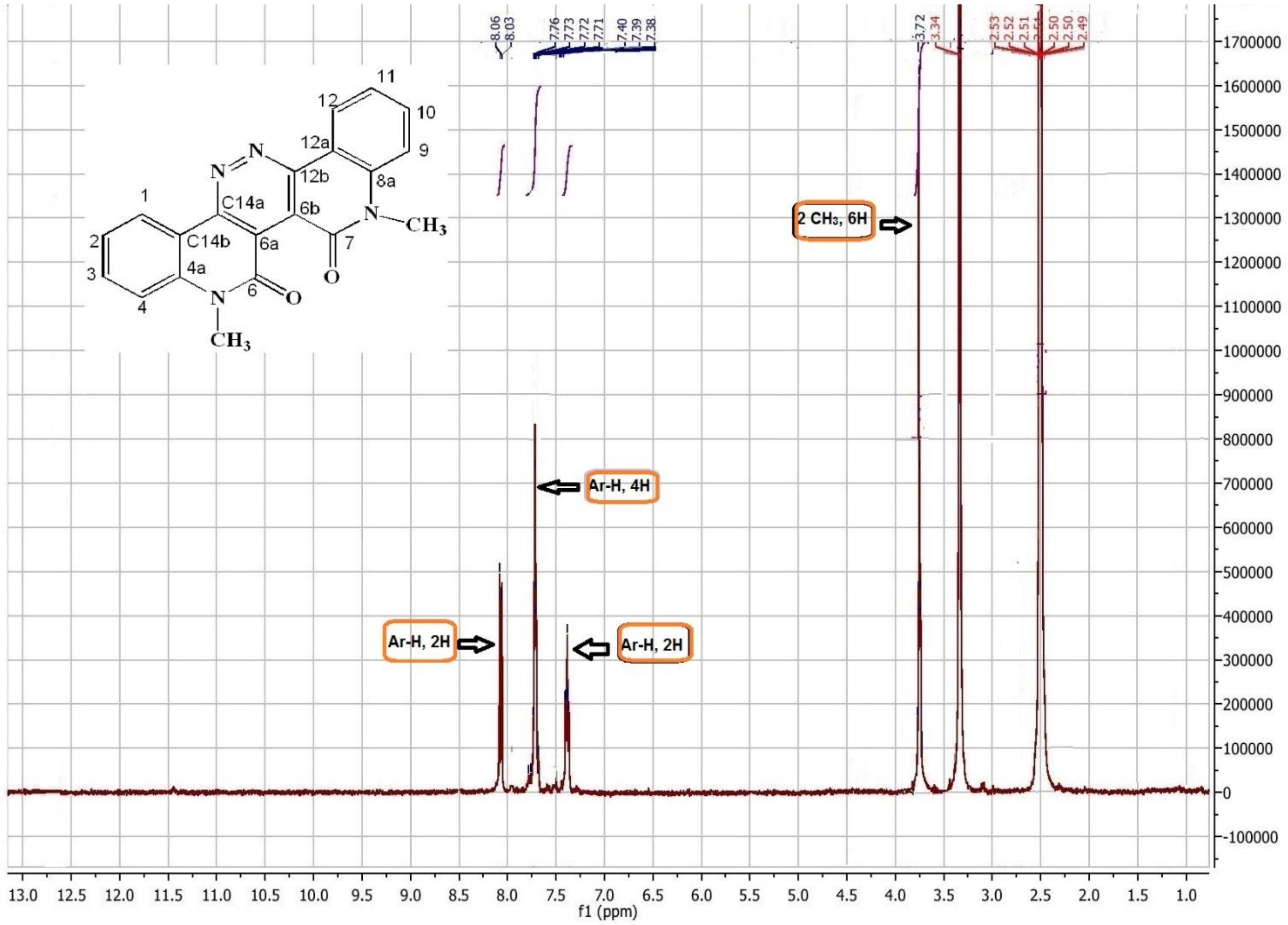

Figure S2: ^{1}H NMR of **3b** (400 MHz, DMSO-d_6, 22 °C**)**

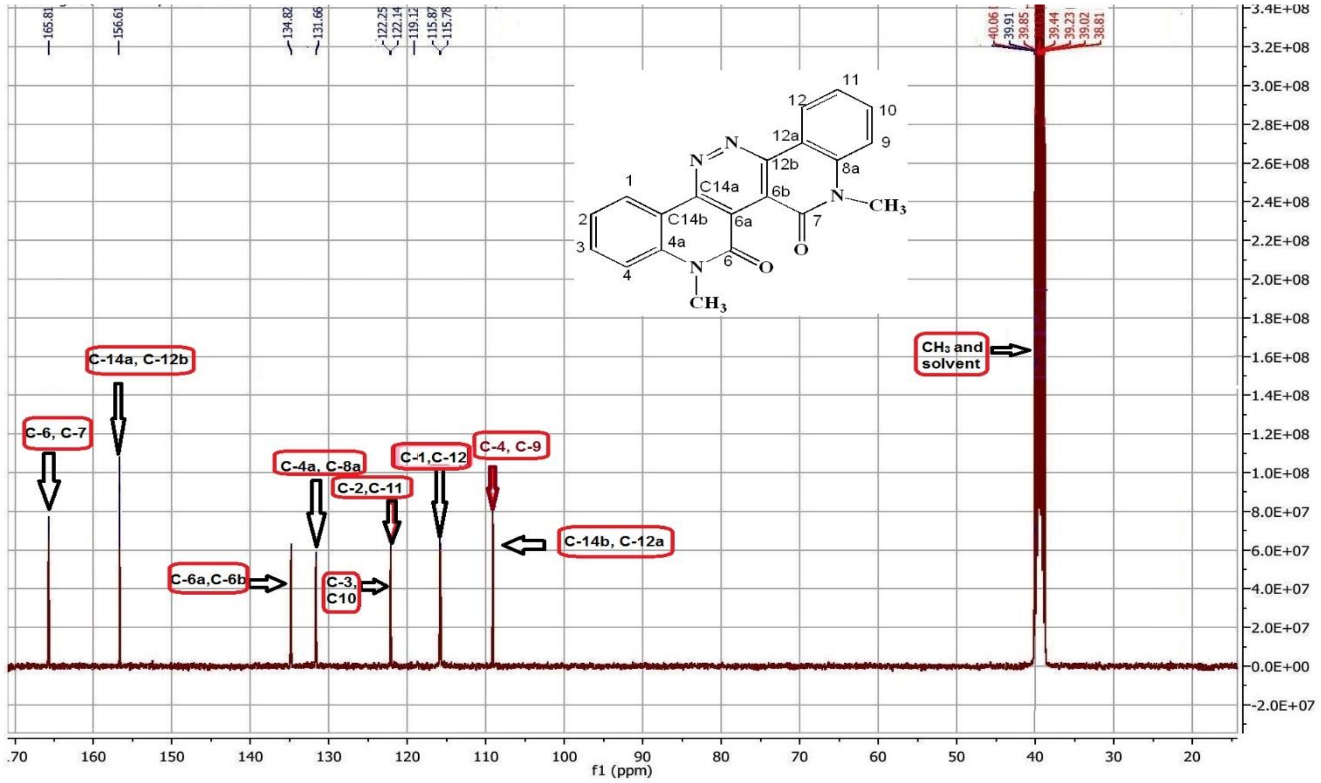

Figure S3: ^{13}C NMR of **3b** (100 MHz, DMSO-d_6, 22 °C**)**

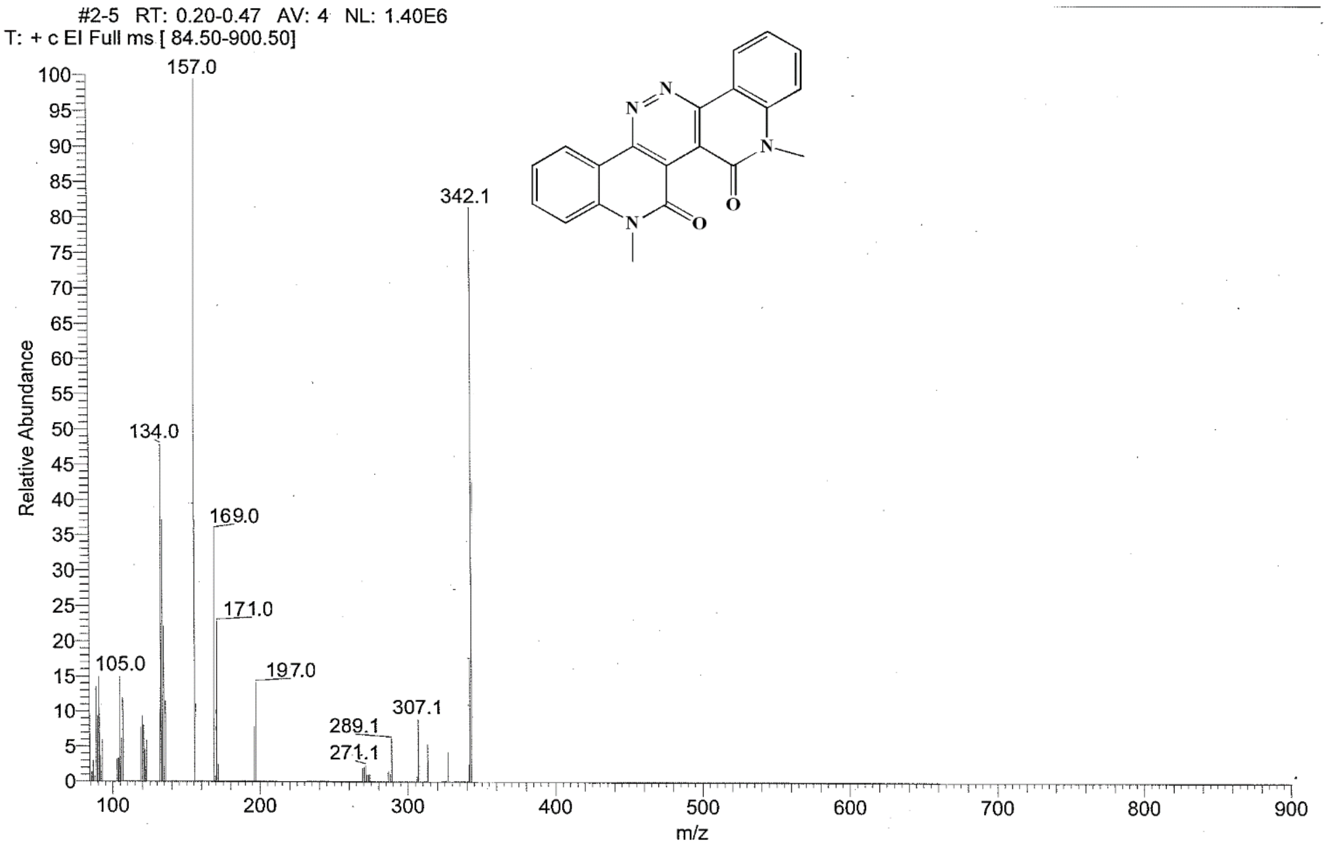

Figure S4: Mass of compound **3b**

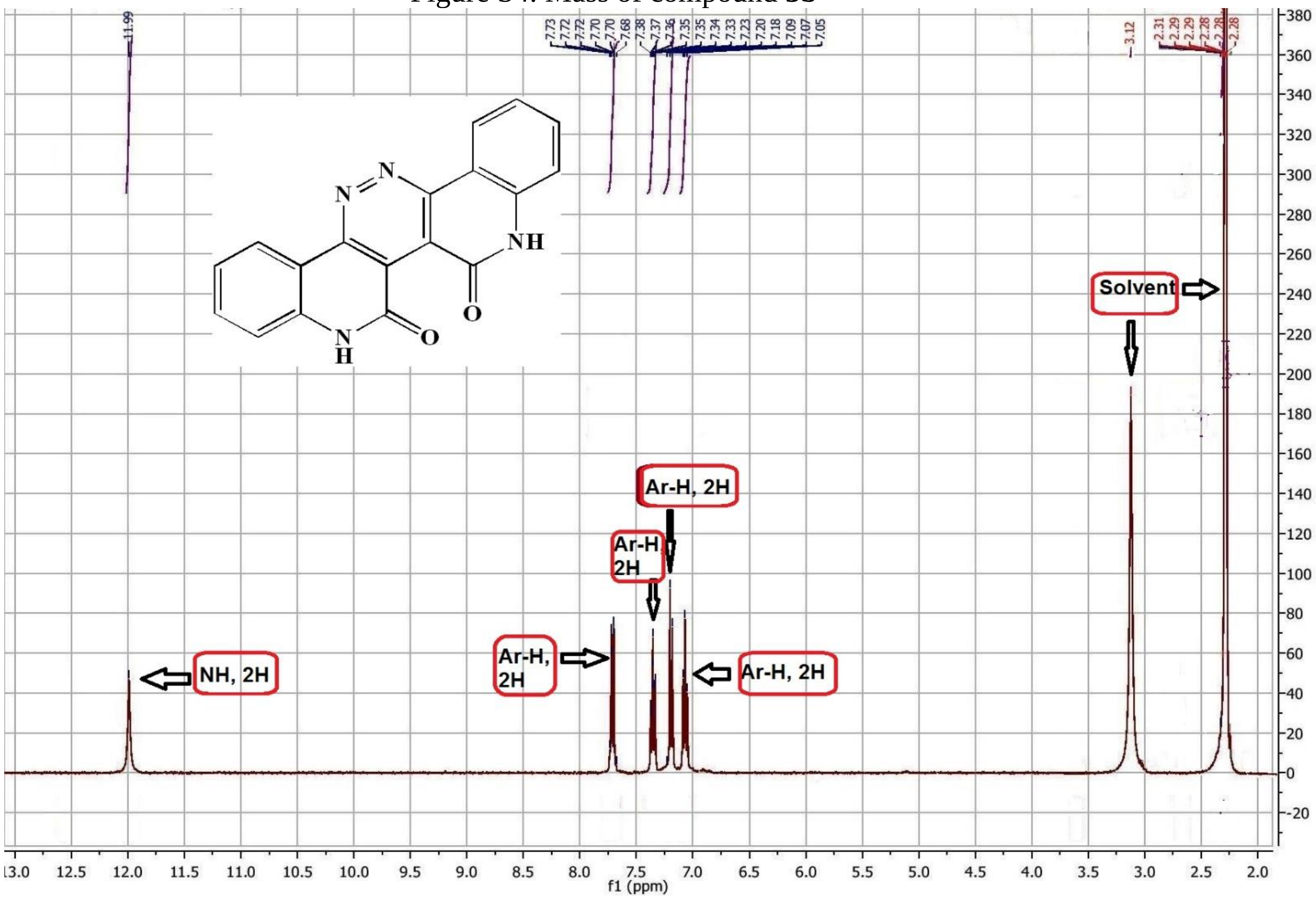

Figure S5: ^{1}H NMR of **3c** (400 MHz, DMSO-d_6, 22 °C)

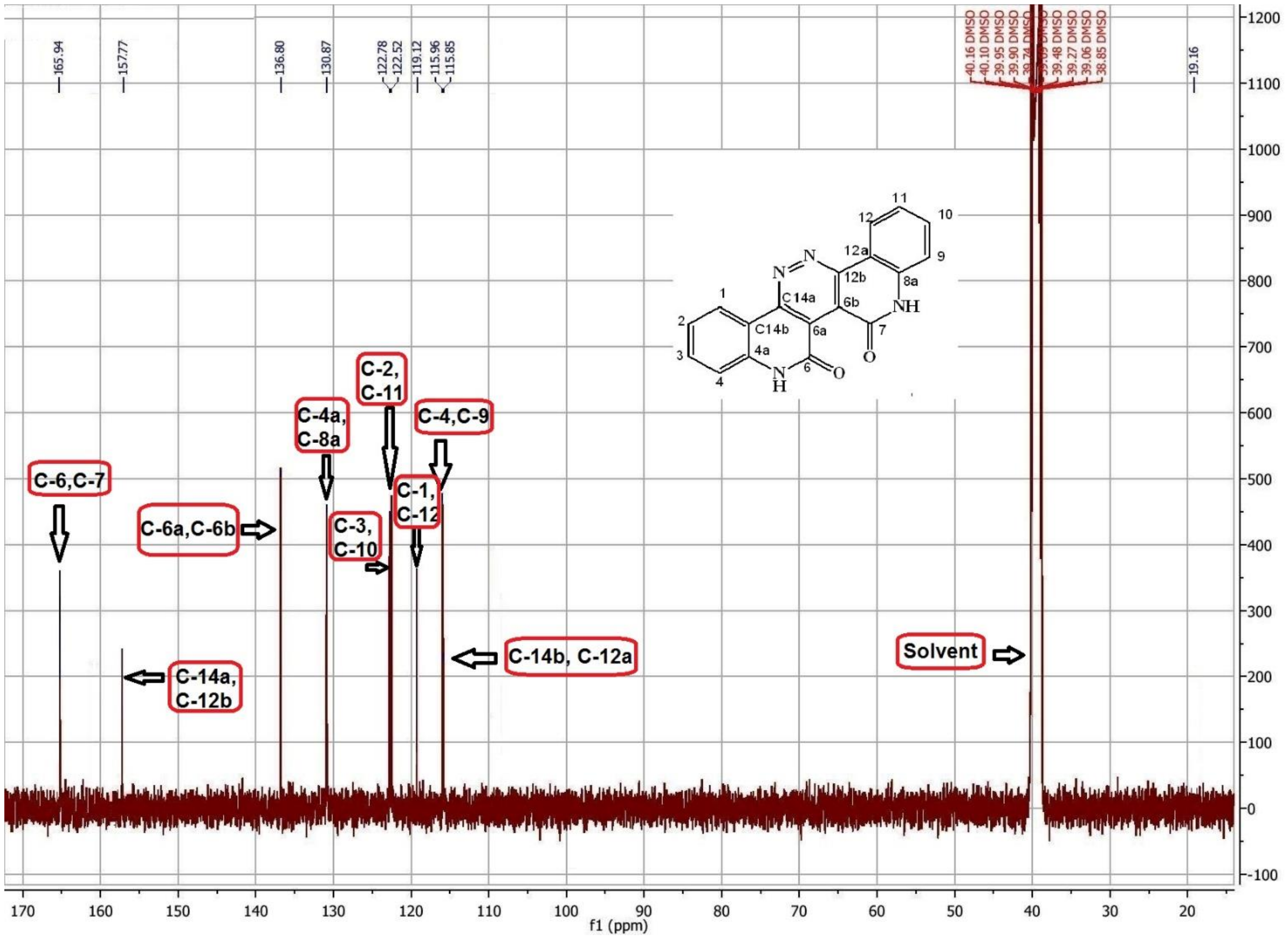

Figure S6: ^{13}C NMR of **3c** (100 MHz, DMSO-d_6, 22 °C**)**

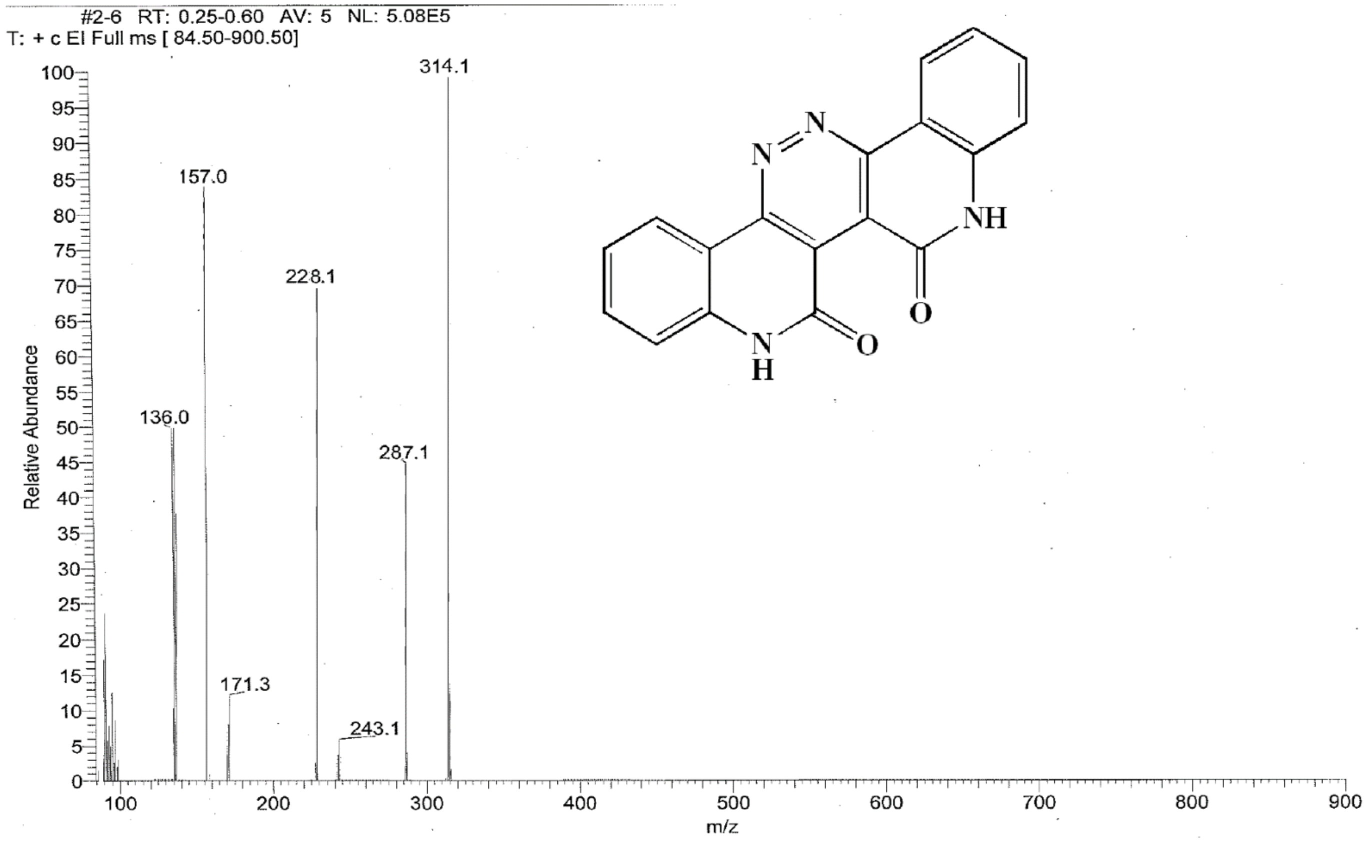

Figure S7: Mass of compound **3c**

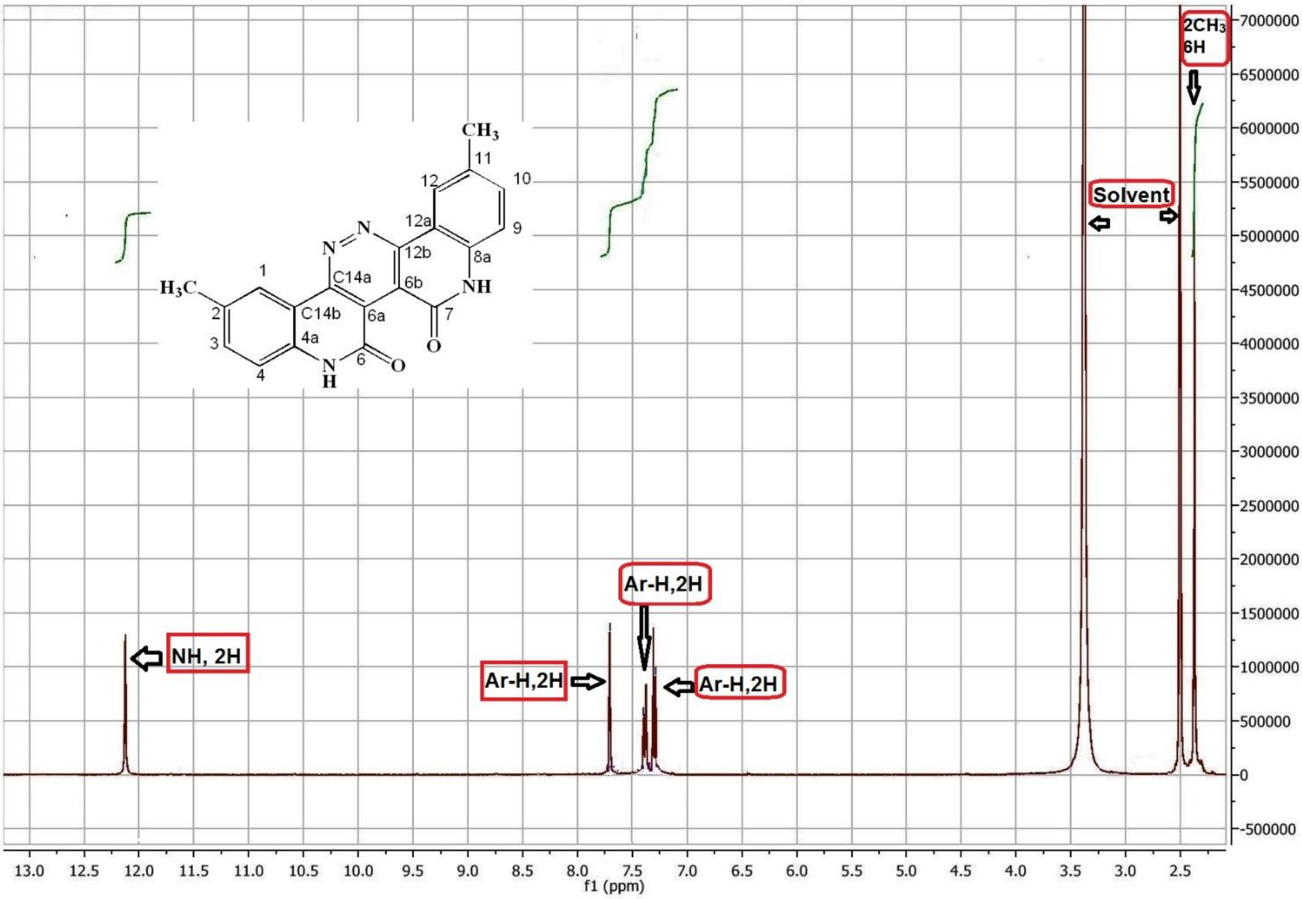

Figure S8: ^{1}H NMR of **3d** (400 MHz, DMSO-d_6, 22 °C**)**

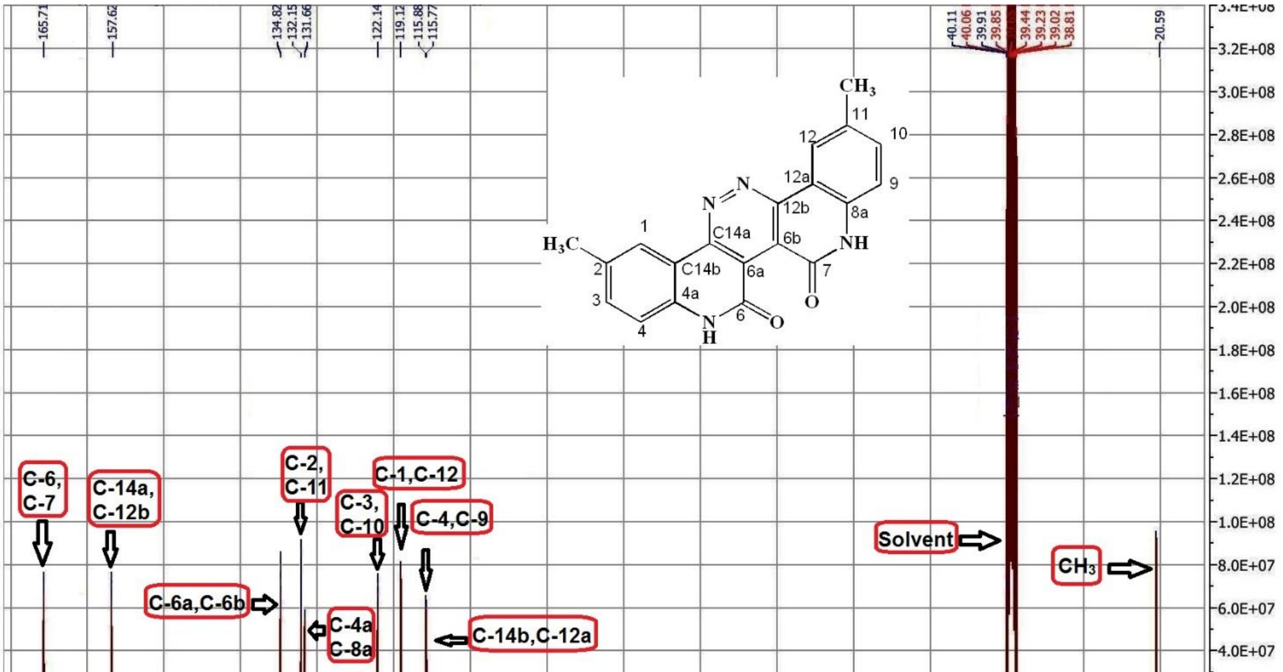

Figure S9: ^{13}C NMR of **3d** (100 MHz , DMSO-d_6, 22 °C**)**

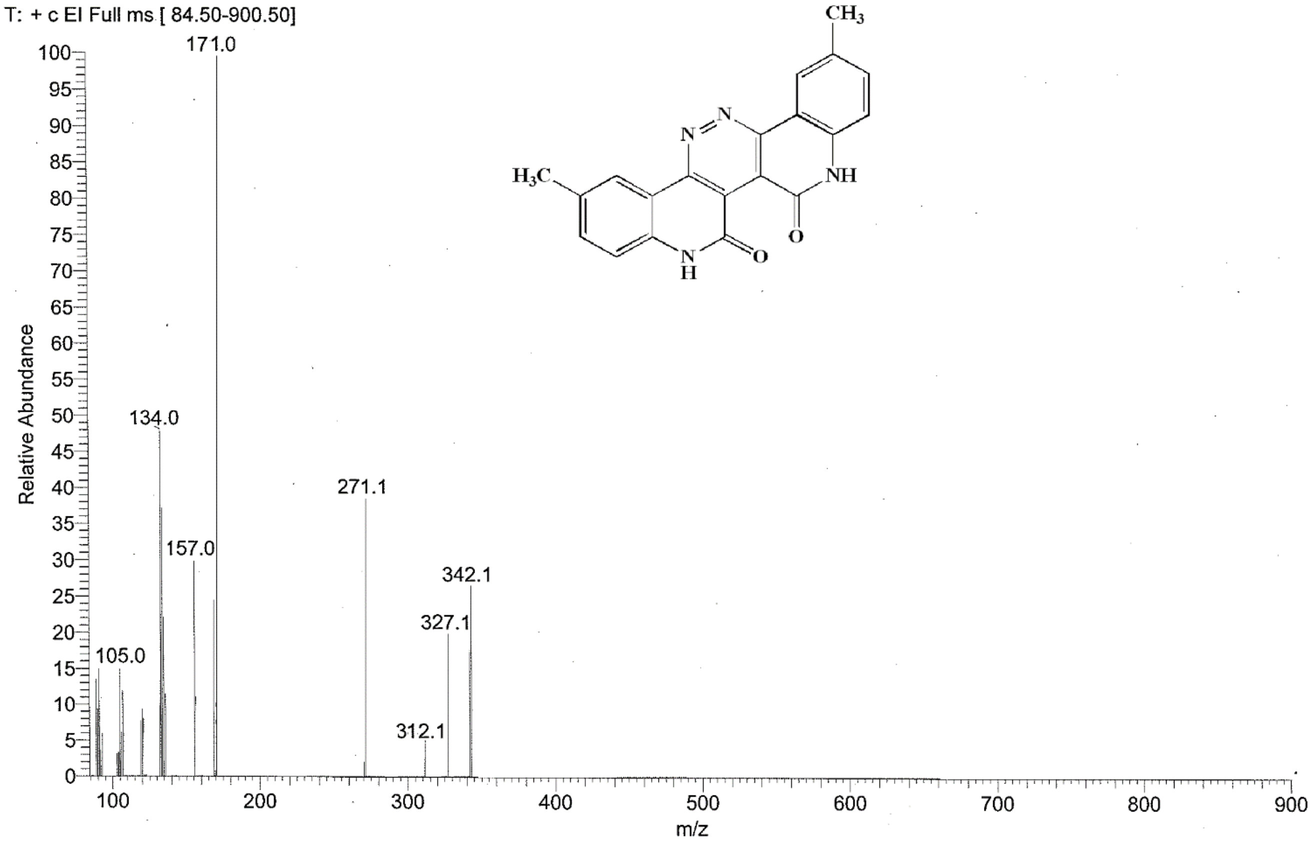

Figure S10: Mass of compound **3d**

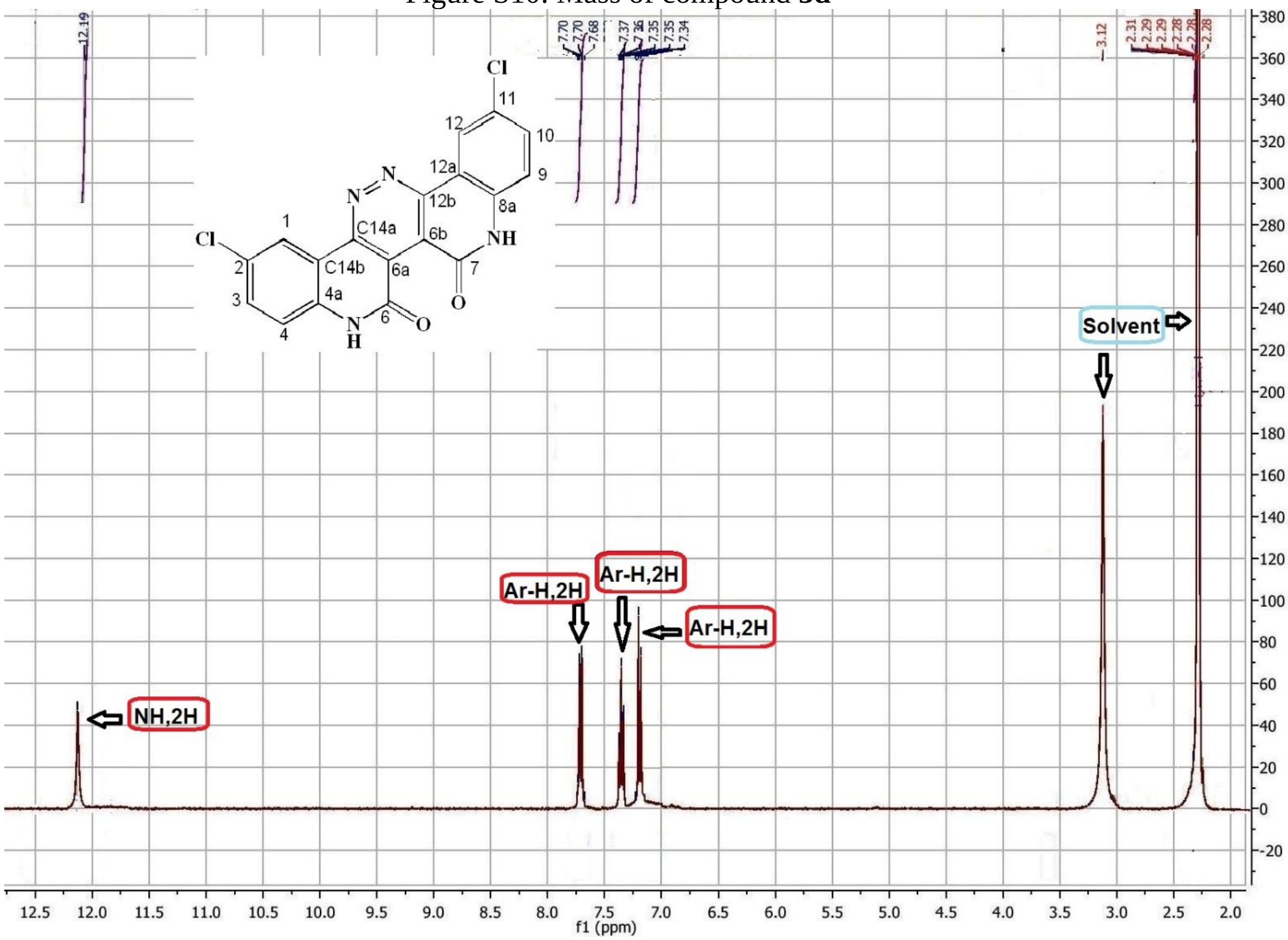

Figure S11: ^{1}H NMR of **3e** (400 MHz, DMSO-d_6, 22 °C)

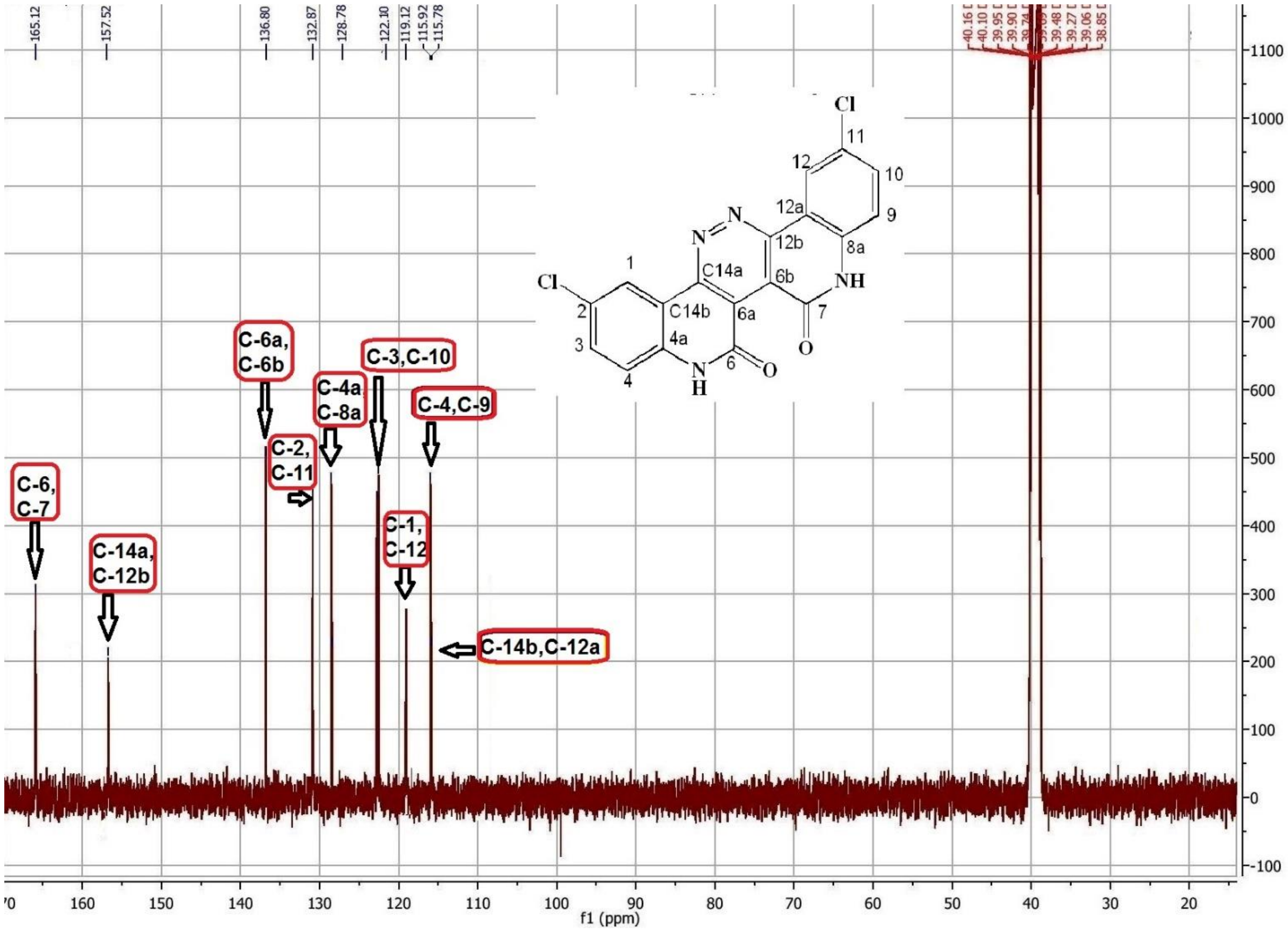

Figure S12: ^{13}C NMR of **3e** (100 MHz , DMSO-d_6, 22 °C)

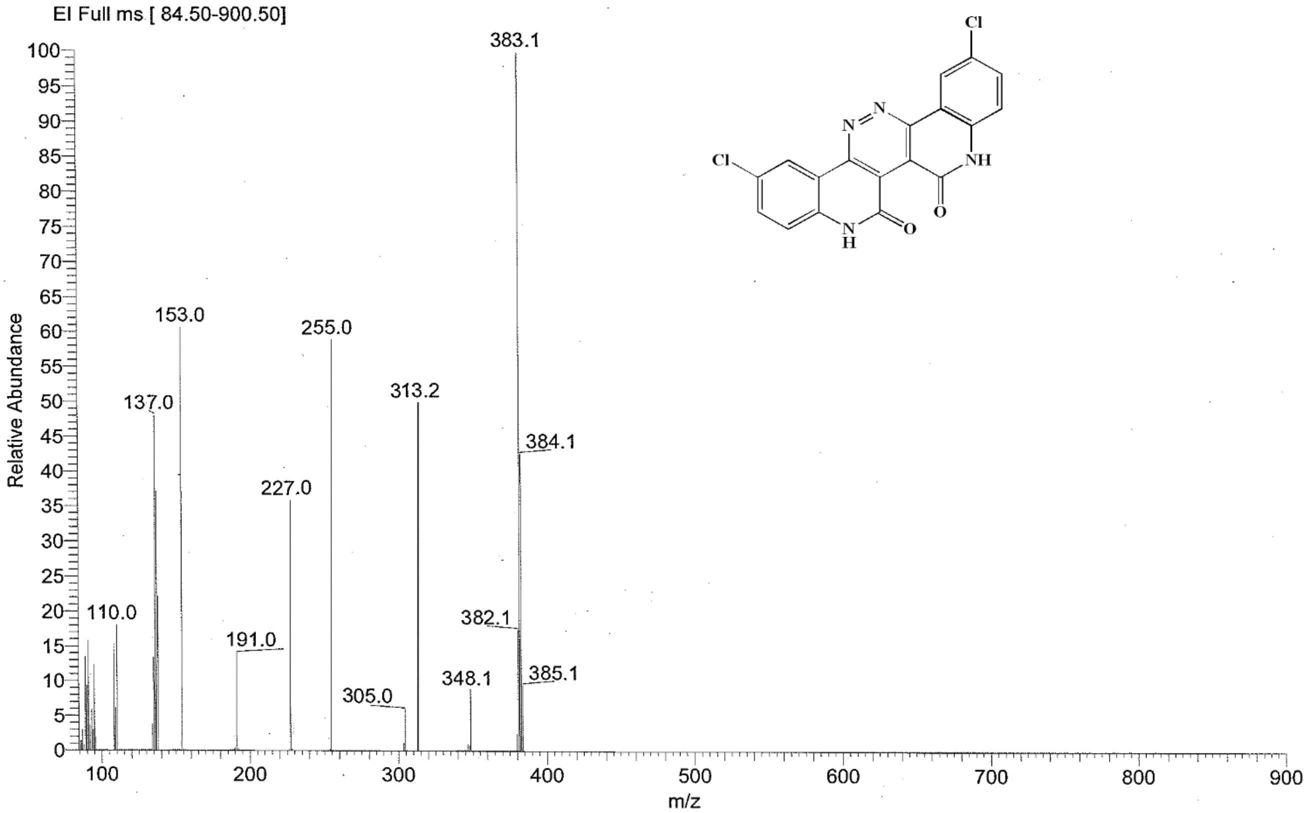

Figure S13: Mass of compound **3e**

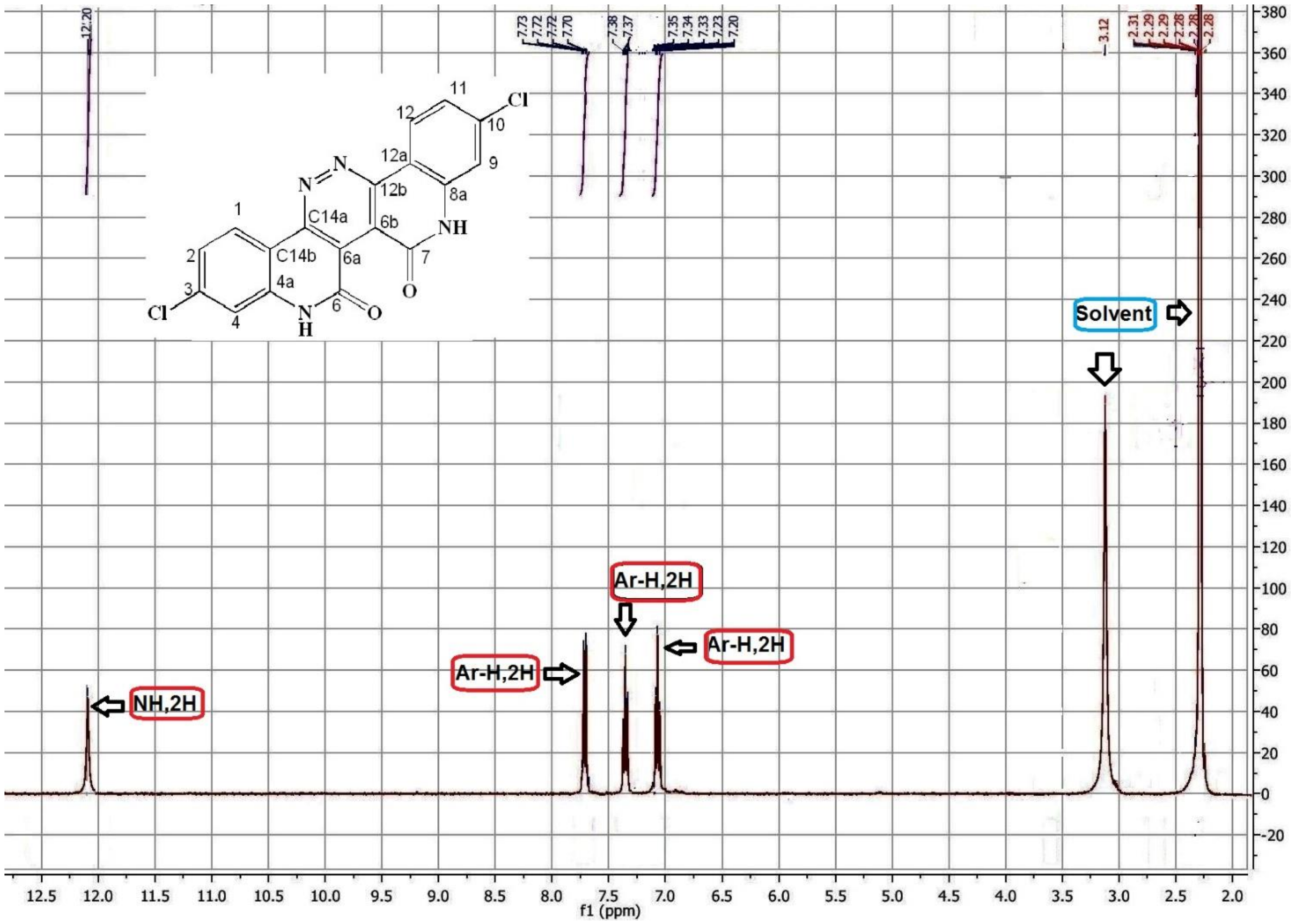

Figure S14: ^{1}H NMR of **3f** (400 MHz, DMSO-d_6, 22 °C)

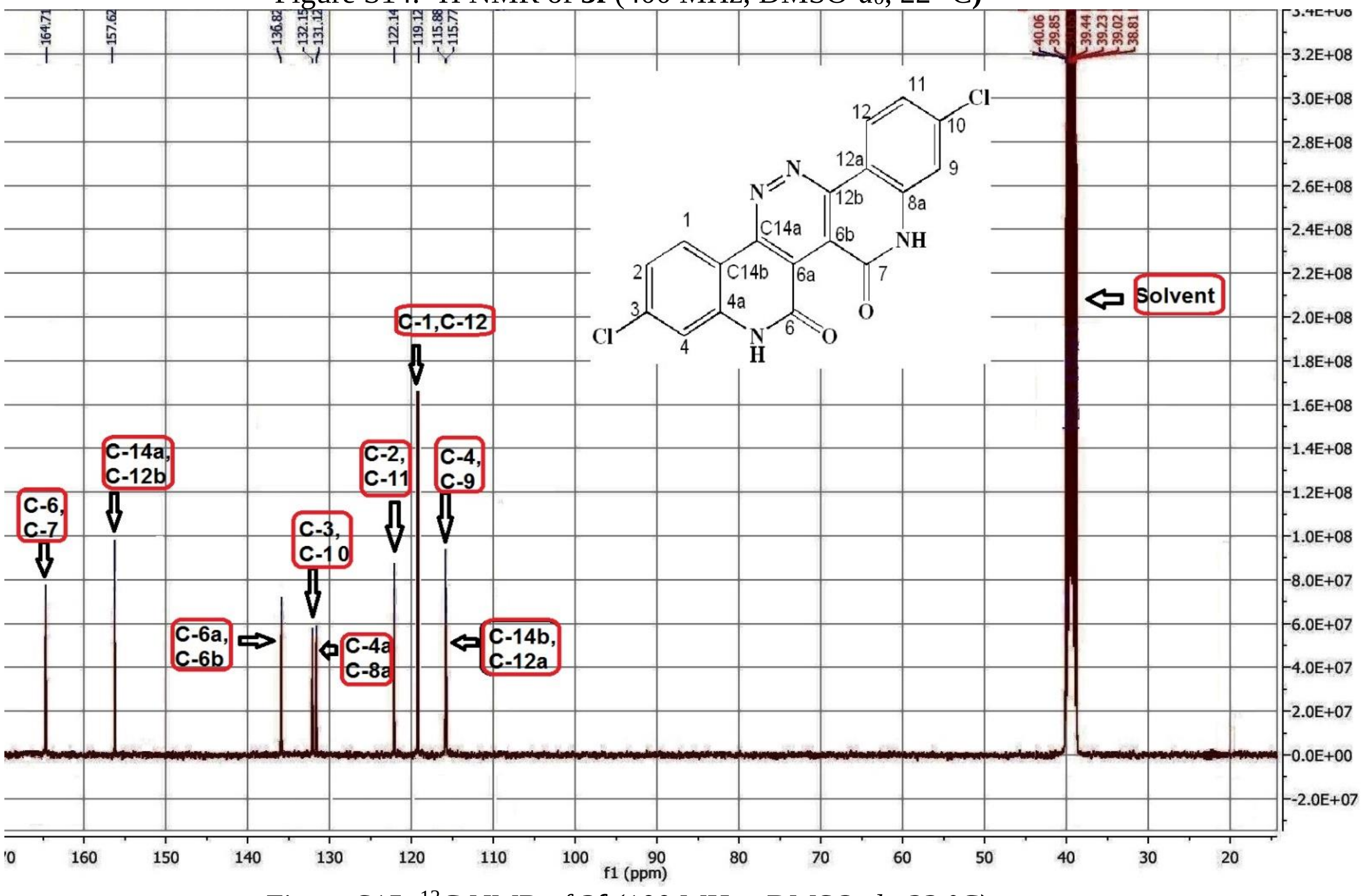

Figure S15: ^{13}C NMR of **3f** (100 MHz , DMSO-d_6, 22 °C)

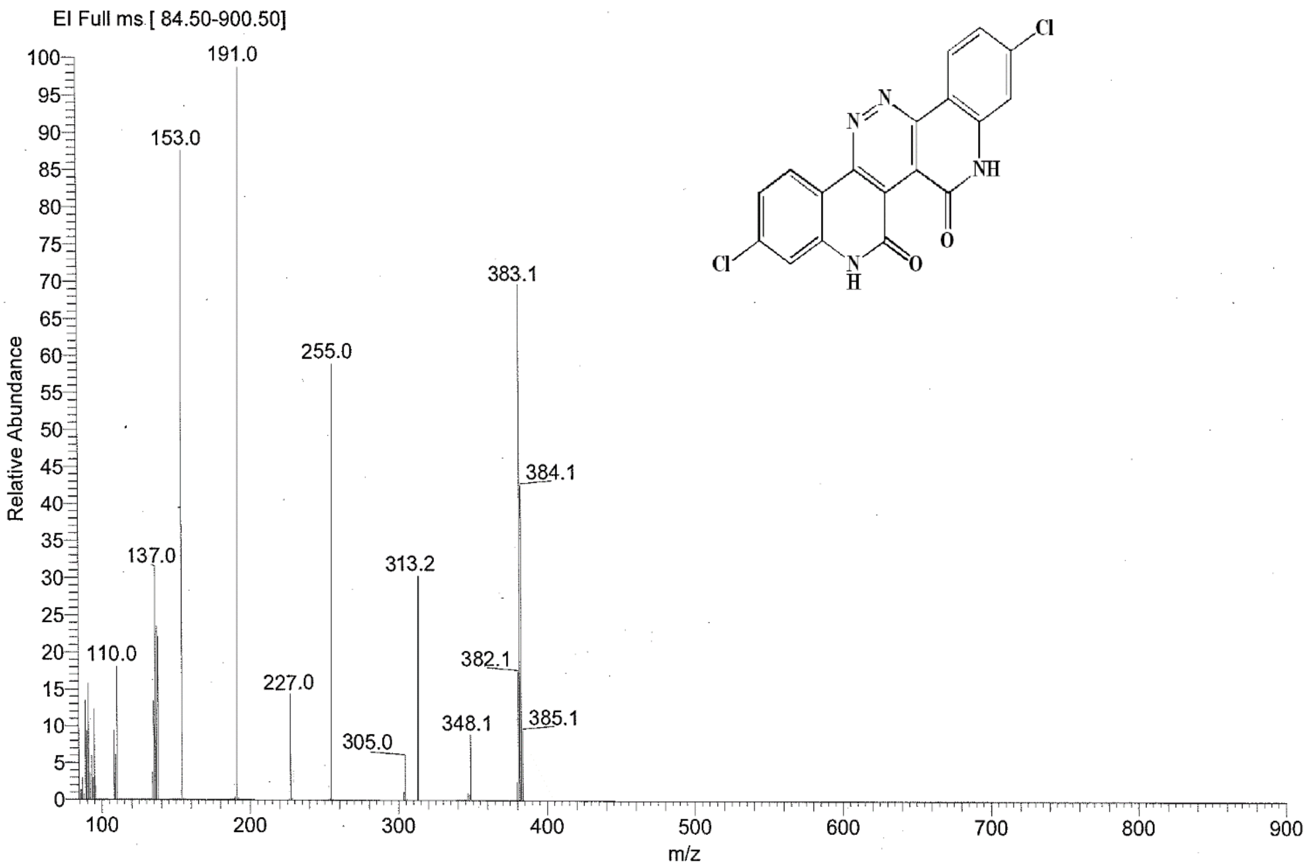

Figure S16: Mass of compound **3f**

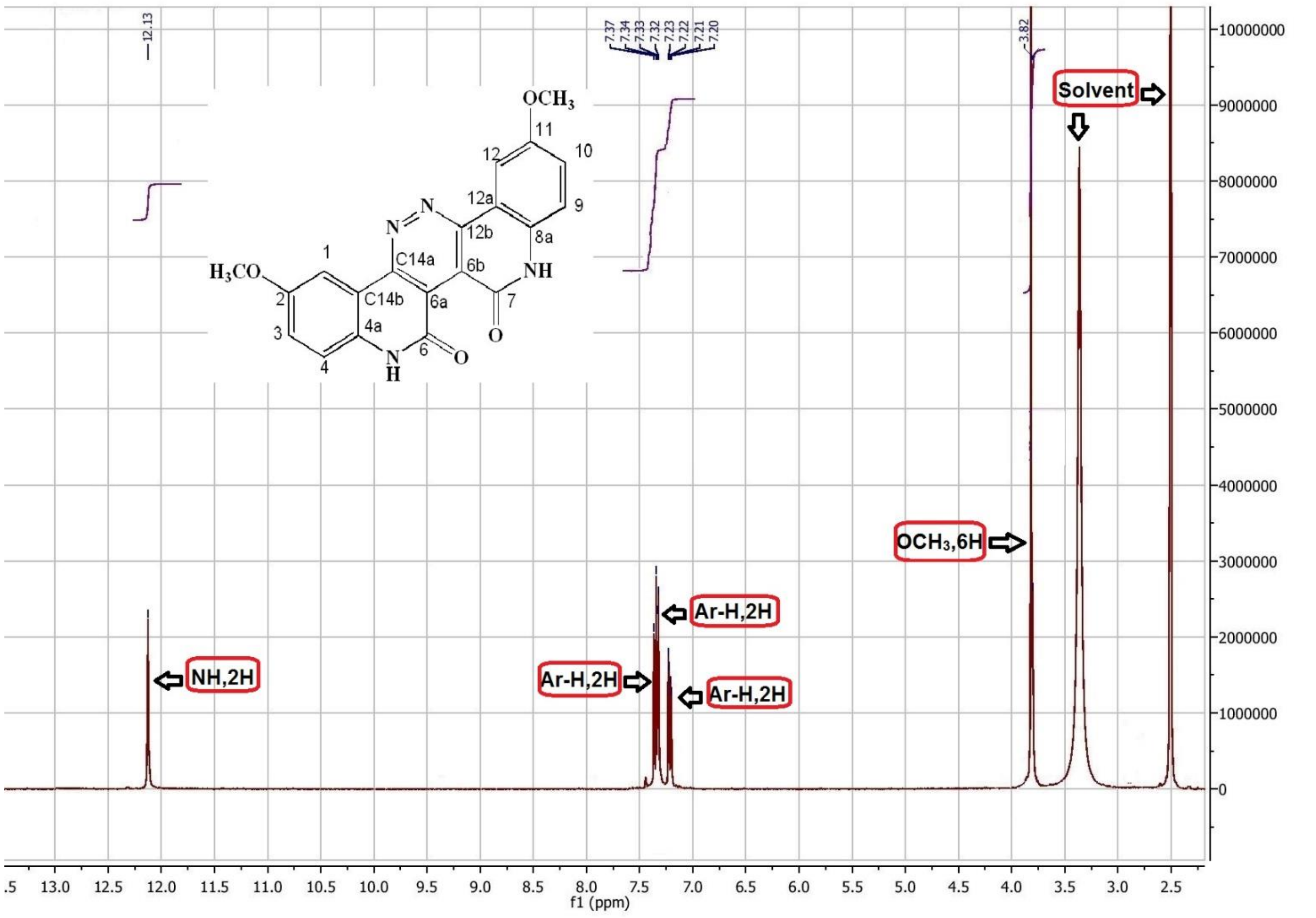

Figure S17: ^{1}H NMR of **3g** (400 MHz, DMSO-d_6, 22 °C)

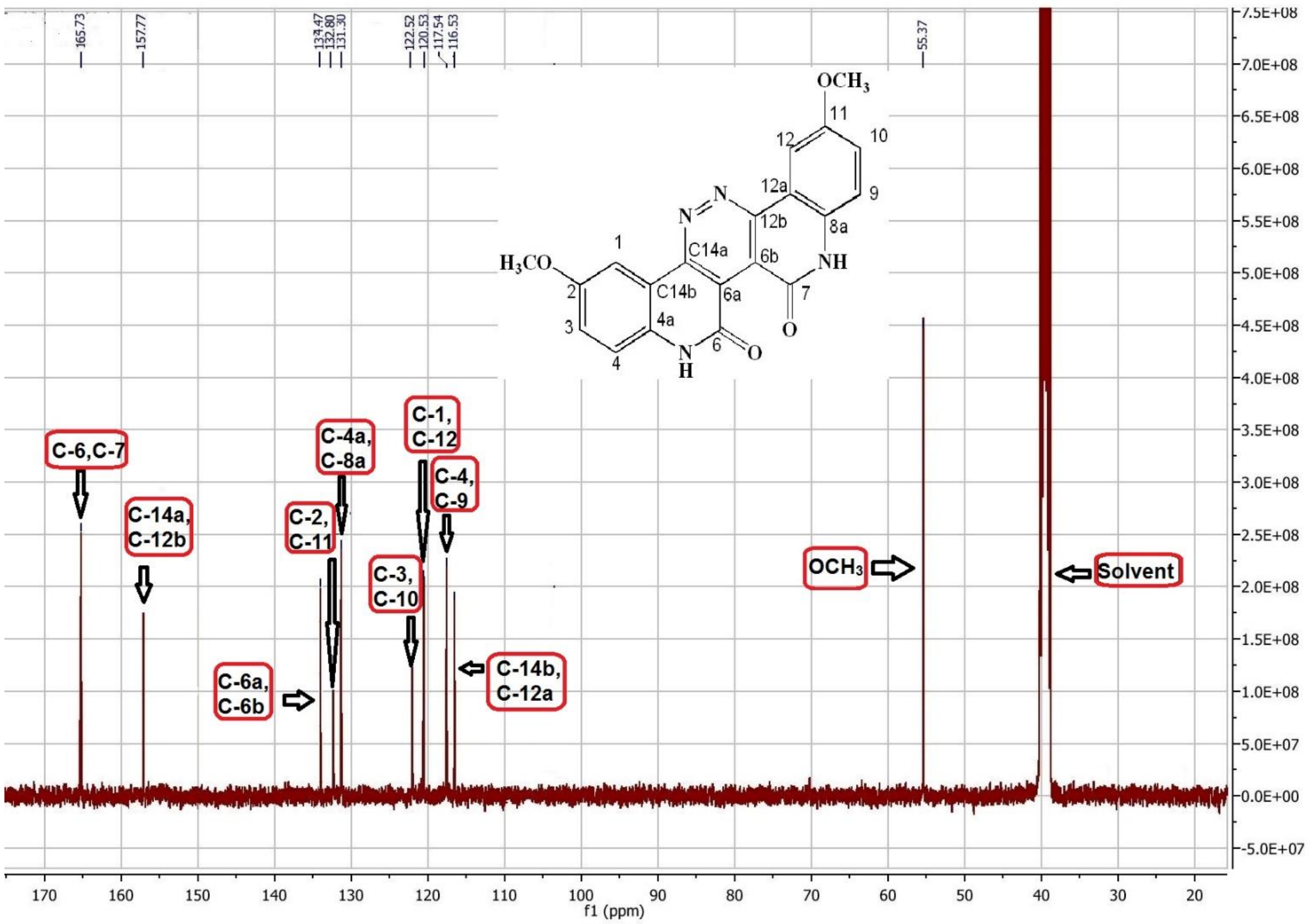

Figure S18: ^{13}C NMR of **3g** (100 MHz , DMSO-d_6, 22 °C)

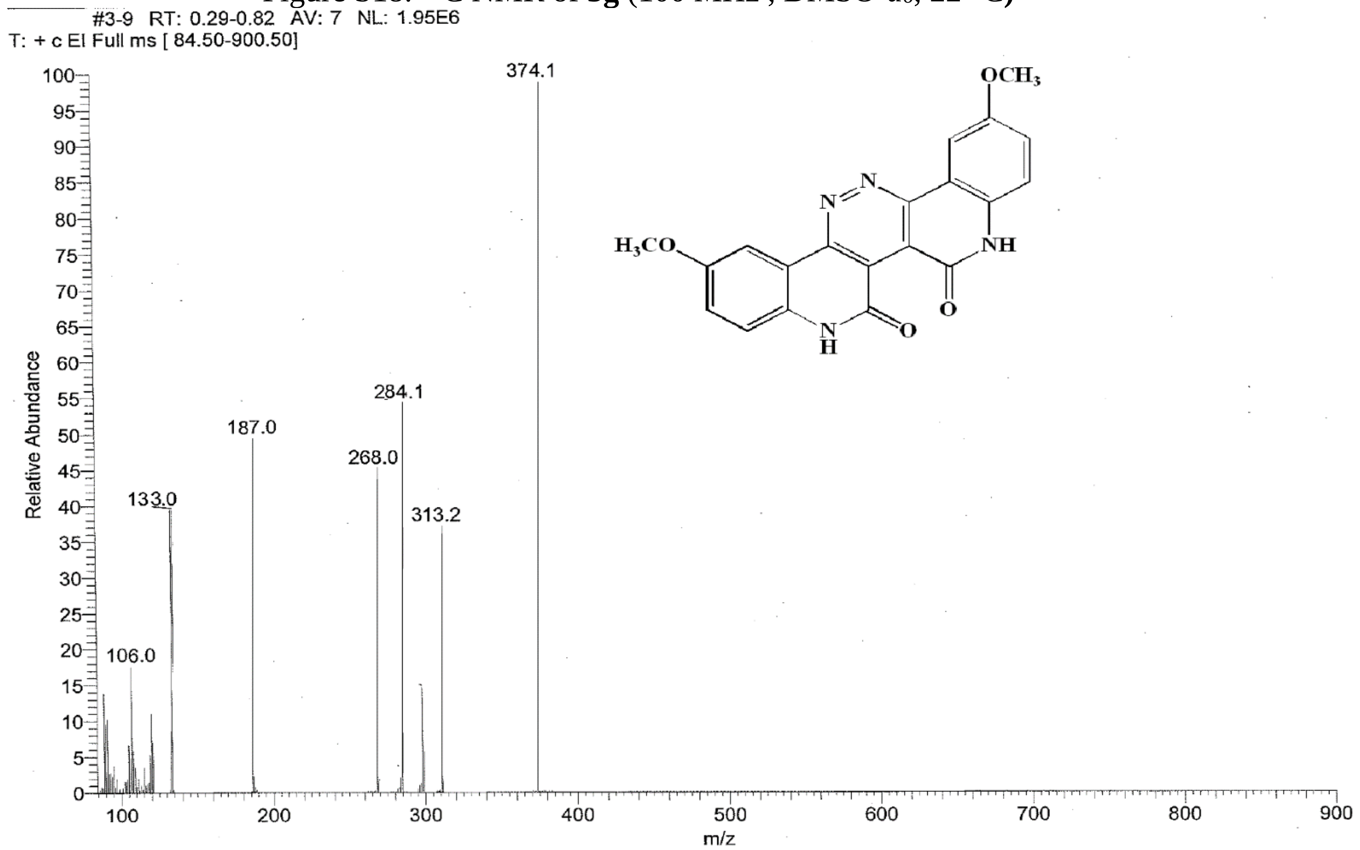

Figure S19: Mass of compound **3g**

Supplementary; Cartesian coordinates of the compound **3a** used in DFT calculation should be informed.

<u>*Compound 3a*</u>

C	5.23678100	10.23465700	4.09975900
H	5.45871400	9.87310000	3.10235200
C	4.16696100	11.08302500	4.33632700
H	3.53325200	11.40682100	3.51641200
C	3.91314500	11.51504900	5.64326900
H	3.07827200	12.18053600	5.84525000
C	4.72173700	11.11092300	6.69831900
H	4.50565000	11.47791100	7.69309900
C	5.81404100	10.25047600	6.47284600
N	6.66749800	9.85756800	7.51465900
C	7.82702400	9.10110000	7.31101700
O	8.72326800	9.08218900	8.14156400
C	7.90784600	8.38807500	6.00734200
C	8.80923200	7.34159000	5.74059600
C	9.40780300	6.47438400	6.79141500
O	8.94167200	6.36561200	7.91582600
N	10.52496400	5.73020500	6.39603100
C	10.85357100	5.49301600	5.05292700
C	11.91563800	4.64074000	4.69242100
H	12.51722800	4.15867800	5.45180300
C	12.19762600	4.39279600	3.35477900
H	13.02151300	3.72988200	3.10491000
C	11.43300200	4.97714700	2.33838700
H	11.65671800	4.77496900	1.29542700
C	10.38635600	5.81940100	2.67803200
H	9.77802400	6.29611400	1.91811200
C	10.08664900	6.09358700	4.02385300
C	9.03236700	7.02651100	4.39114100
N	8.31855700	7.59778500	3.38367500
N	7.36138900	8.44034800	3.64935200
C	7.14979000	8.86213900	4.92536800
C	6.06415900	9.80393600	5.15150700
C	6.52361300	10.42461300	8.86861300
H	7.03311200	9.73215300	9.53883700
H	5.46152600	10.41760700	9.12635500
C	7.13303900	11.82101700	9.00971300
H	6.98803800	12.18795600	10.03208200
H	8.20706900	11.77991800	8.80901700
H	6.67484900	12.53812300	8.32083400
C	11.20548500	5.00280400	7.48382700
H	11.04240500	5.59251100	8.38601500
H	12.27783300	5.00588200	7.27267900
C	10.67161100	3.58402800	7.69194000
H	11.22032600	3.09636700	8.50557300
H	9.61369400	3.62139000	7.96564500
H	10.77925500	2.97086100	6.79129000

Compound 3'a

C	4.52764900	10.24415400	5.82686900
H	4.54426300	9.81437900	4.83242700
C	3.53507500	11.12903500	6.21473400
H	2.74942200	11.40879200	5.51970700
C	3.55979100	11.66135000	7.50936700
H	2.79165200	12.36169000	7.82579100
C	4.56339100	11.31426000	8.40466000
H	4.56085200	11.75994200	9.39054200
C	5.57404000	10.40921000	8.02469500
N	6.61363000	10.05429300	8.89489400
C	7.66717500	9.20620500	8.54296000
O	8.65661200	9.12480200	9.26734600
C	7.53458200	8.47134700	7.25778100
C	8.41539900	7.43410700	6.82930200
C	9.46785600	6.41204200	4.76043800
O	9.55446500	6.30702700	3.54600800
N	10.42038900	5.84029000	5.60631400
C	10.26272200	5.78408600	6.99427900
C	11.07968500	4.93522000	7.76736800
H	11.84813900	4.33960900	7.29326300
C	10.88236200	4.81042300	9.13435900
H	11.51926400	4.13795300	9.70252300
C	9.85455000	5.51467500	9.76838500
H	9.67222900	5.39088200	10.83120000
C	9.06414900	6.37470500	9.02576500
H	8.27074300	6.91287800	9.52062300
C	9.26459000	6.56714200	7.64366700
C	8.40352100	7.20274300	5.43777700
N	7.48189500	7.70792500	4.59273100
N	6.53182600	8.47915700	5.04153000
C	6.57608700	8.91486500	6.32923100
C	5.54880800	9.86878100	6.71883900
C	6.74148000	10.70095300	10.21536000
H	7.33990200	10.02600700	10.82608000
H	5.74542200	10.76076400	10.66013100
C	7.41697700	12.07179100	10.14893600
H	7.47617400	12.50637600	11.15309900
H	8.43386900	11.96849700	9.75995400
H	6.86618700	12.76693700	9.50702500
C	11.54633900	5.17385900	4.92580800
H	12.42855300	5.26791100	5.56316800
H	11.72699400	5.74748600	4.01612000
C	11.25765800	3.71636500	4.55877100
H	12.12914300	3.28016200	4.05761600
H	11.02902500	3.10765600	5.43979400
H	10.40849400	3.66808300	3.87186600
